# *GRN* rs5848 variant associates with TDP-43 pathology and cancer in opposite directions

**DOI:** 10.1093/jnen/nlag051

**Published:** 2026-05-21

**Authors:** Khine Zin Aung, Peter T Nelson, Xiaotong Ning, Xian Wu, Inori Tsuchiya, Shama Karanth, Erin L Abner, David W Fardo, Yuriko Katsumata

**Affiliations:** Division of Biomedical Informatics, Department of Internal Medicine, University of Kentucky, Lexington, KY, United States; Sanders-Brown Center on Aging, University of Kentucky, Lexington, KY, United States; Sanders-Brown Center on Aging, University of Kentucky, Lexington, KY, United States; Department of Pathology, University of Kentucky, Lexington, KY, United States; Sanders-Brown Center on Aging, University of Kentucky, Lexington, KY, United States; Department of Epidemiology and Environmental Health, University of Kentucky, Lexington, KY, United States; Division of Biomedical Informatics, Department of Internal Medicine, University of Kentucky, Lexington, KY, United States; Sanders-Brown Center on Aging, University of Kentucky, Lexington, KY, United States; Sanders-Brown Center on Aging, University of Kentucky, Lexington, KY, United States; Department of Biostatistics, University of Kentucky, Lexington, KY, United States; Department of Surgery, College of Medicine, University of Florida, 1600 SW Archer Rd, Gainesville, FL, 32608, United States; UF Health Cancer Center, University of Florida, 2033 Mowry Rd, Gainesville, FL, 32610, United States; Sanders-Brown Center on Aging, University of Kentucky, Lexington, KY, United States; Department of Epidemiology and Environmental Health, University of Kentucky, Lexington, KY, United States; Sanders-Brown Center on Aging, University of Kentucky, Lexington, KY, United States; Department of Biostatistics, University of Kentucky, Lexington, KY, United States; Division of Biomedical Informatics, Department of Internal Medicine, University of Kentucky, Lexington, KY, United States; Sanders-Brown Center on Aging, University of Kentucky, Lexington, KY, United States

**Keywords:** Apolipoprotein E, epidemiology, FTLD-TDP, LATE-NC, neurodegeneration, NIAGADS, rs6733839, rs744373

## Abstract

Epidemiologic studies have reported that cancer survivors have a relatively low risk of developing dementia, but the mechanisms underlying that inverse relationship are mostly unknown. The Granulin (*GRN*) gene single nucleotide variant rs5848 T allele is associated with increased risk of limbic-predominant age-related TDP-43 encephalopathy neuropathologic change (LATE-NC) and hippocampal sclerosis of aging (HS-Aging). The T allele is also associated with lower expression of the cognate protein progranulin (PGRN), which is a mitogen implicated in neoplasia. We examined whether the rs5848 variant associated with LATE-NC/HS-Aging pathology and cancer in the same cohort. This study leveraged genotype data from the Alzheimer’s Disease Genomics Consortium (*n = *8121) and the Alzheimer’s Disease Sequencing Project (*n = *3231), with cancer history and neuropathology data drawn from the National Alzheimer’s Coordinating Center. The rs5848 T allele was associated with higher odds of LATE-NC (*p < *0.001) and was also associated with lower odds of cancer (*p = *0.012). Established *TMEM106B*, *APOE*, and *BIN1* risk alleles for Alzheimer’s disease showed no associations with cancer, implying that the *GRN*-related associations could not be completely explained by selection bias in the study sample. The finding of a specific allele with opposite correlative impact on cancer risk and dementia-related pathology has potential therapeutic implications.

## Introduction

Epidemiologic and observational studies have reported an inverse relationship between cancer and dementia such that individuals with a history of cancer have a relatively low risk of developing dementia, while those with dementia are comparatively unlikely to be diagnosed with cancer.^1–6^ Methodological factors may contribute to the detected phenomena, with potential underlying confounders including survival and surveillance biases as patients with cancer may die before developing dementia (and vice versa). Moreover, study inclusion criteria, health-care utilization and volunteer participation may differ across observational cohorts. In addition to study design and related sources of bias, biological mechanisms may underlie this relationship. Treatments commonly used in cancer care, such as chemotherapies that affect neuroinflammation and endocrine function, may modify dementia risk.^7–9^ In addition, constitutive biological pathways that drive neurodegeneration or tumorigenesis may suppress the other.

Neurodegenerative diseases and cancer are influenced by overlapping biological pathways involving protein homeostasis, inflammatory regulation, and cell growth.^1^^,^^4^^,^^7^^,^^10^ Among genes implicated in human diseases, granulin (*GRN*) is distinctive in being directly linked to both dementia and cancer. *GRN* plays key roles in normal cellular function and brain health and its encoded protein progranulin (PGRN), is involved in lysosomal activity, inflammation, and cell proliferation.^11^^,^^12^ Increased levels of PGRN are mitogenic and PGRN is recognized as an oncogenic marker in several cancers.^13^ Conversely, PGRN deficiency promotes the accumulation of TDP-43 pathology, a hallmark of frontotemporal lobar degeneration with TDP-43 inclusions (FTLD-TDP) and limbic-predominant age-related TDP-43 encephalopathy neuropathologic change (LATE-NC); it exacerbates lysosomal dysfunction, neuroinflammation and microglial hyperactivation thereby apparently contributing to neuronal damage and cognitive decline.^14–16^

The *GRN* rs5848 single-nucleotide polymorphism (SNP) is a notable variant located in the 3'-untranslated region (UTR) of *GRN*. The T allele of rs5848 is associated with reduced PGRN expression in both brain and blood.^17–20^ In numerous studies, this SNP has been implicated in neurodegenerative conditions such as LATE-NC and hippocampal sclerosis of aging (HS-Aging), and the T allele has also been reported as a risk factor for younger age-at-onset in FTLD-TDP.^17^^,^^21–26^ Although existing studies have not directly examined the association of rs5848 with cancer (with the exception of one study in a cohort of persons with Parkinson disease^27^) the potential effects of PGRN expression decreasing rs5848 can be inferred from the role of *GRN* in cancer biology, as elevated PGRN levels promote tumor growth and function as a mitogenic factor.^11^

**Figure 1:**
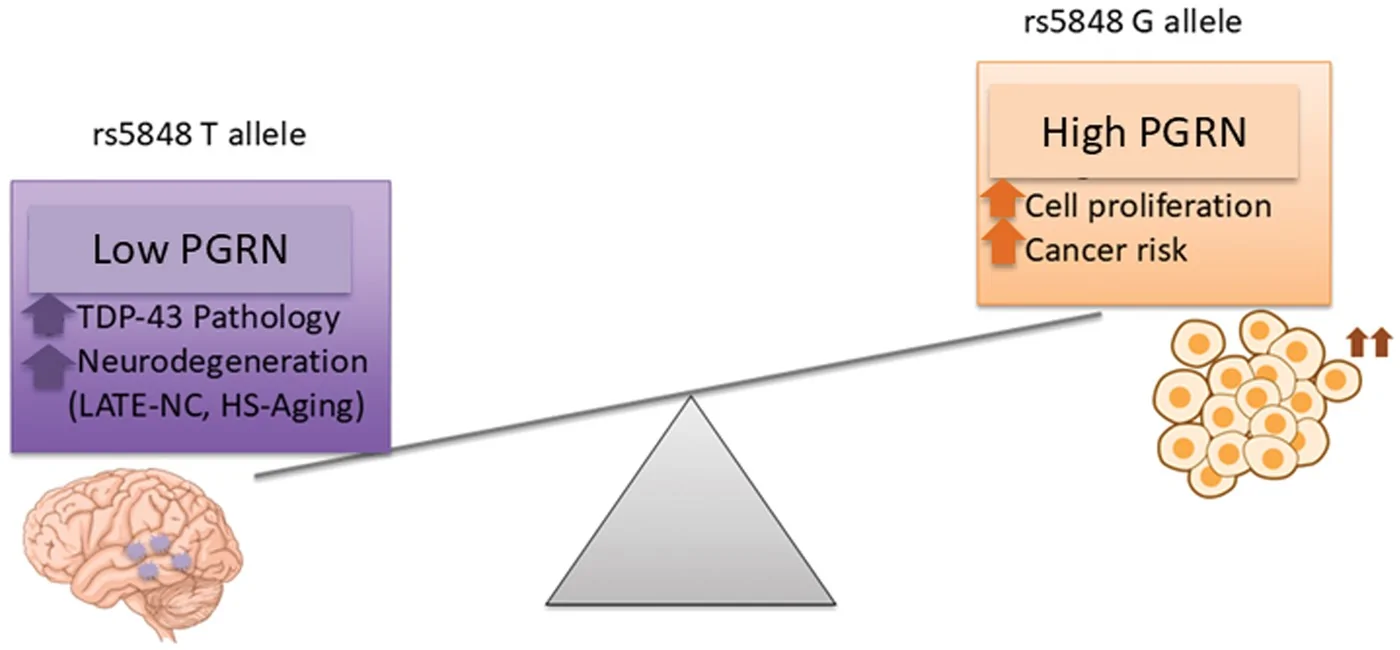
Biological trade-off model of *GRN* rs5848. PGRN: progranulin, LATE-NC: limbic-predominant age-related TDP-43 encephalopathy neuropathologic change, HS-aging: hippocampal sclerosis of aging.

These observations suggest that the T allele of rs5848, which is associated with increased dementia risk may theoretically exert a protective influence against cancer development or progression. Therefore, we investigated whether rs5848 was associated with cancer status as well as TDP-43–related neuropathologic outcomes, focusing on autopsy-confirmed LATE-NC and HS-Aging. We also conducted parallel analyses of rs1990622, located in *TMEM106B*, which is a known genetic risk SNP for LATE-NC and HS-Aging, as well as established Alzheimer disease (ad) risk alleles in Apolipoprotein E (*APOE;* ε4), and two bridging integrator 1 (*BIN1*) risk SNPs (rs744373 and rs6733839). These comparisons allowed us to test the hypothesis that the inverse relationship between cancer and neurodegenerative disease was partly attributable to the *GRN* rs5848 variant. We therefore hypothesize a biological trade-off model whereby *GRN* rs5848 influences neurodegeneration and proliferative pathways through differential progranulin expression (Figure 1). llustrations were adapted using BioArt resources provided by the National Institutes of Health (NIH) (https://bioart.niaid.nih.gov).

## Methods

### Study population and data sources

As all study participants were de-identified and the data were available to the public for secondary analyses after an application process, the University of Kentucky Institutional Review Board (IRB) classified this project as not Human Subjects Research under National Institute on Health (NIH) Exemption #4 (https://grants.nih.gov/policy-and-compliance/policy-topics/human-subjects/research).

Clinical and neuropathological data were drawn from the National Alzheimer’s Coordinating Center (NACC) Uniform Data Set (UDS) and Neuropathology (NP) Data Set from the June 2025 data freeze (https://naccdata.org).^28^^,^^29^ Data were collected from 46 Alzheimer’s Disease Research Centers (ADRCs) across the U.S. National Institute on Aging (NIA)–funded ADRC network. All participating centers received IRB approval at their local site and obtained written informed consent from participants.

Cancer history was ascertained from the NACC UDS version 3.0 (March 2015), using Form D2 (Clinician-Assessed Medical Conditions), in which cancer status in the 12 months prior to the UDS visit was clinician-determined.^30^ Participants were considered positive for cancer history based on at least one instance of clinician-reported cancer (excluding non-melanoma skin cancer) across UDS follow-up.

For analyses involving NP outcomes, participants diagnosed with any of 27 rare brain diseases (Supplementary Table 1, available at *Journal of Neuropathology & Experimental Neurology* online) were excluded as previously described;^31^ this mitigated biases inherent to clinic-based cohorts that are enriched for uncommon neurodegenerative disorders relatively affecting younger (middle-aged) individuals.^32^

### Cancer and neuropathological outcomes

Cancer status was coded as: 1 for individuals with a cancer history (as defined above) and 0 for individuals with no reported cancer history.^30^ TDP-43-related pathologies were coded as 0 = absent or 1 = present in each of the amygdala, hippocampus, and neocortex. Pathologically diagnosed HS-Aging, including cases recorded as having mesial temporal sclerosis (MTS), was coded as 0 = absent or 1 = present. ad neuropathologic change (ADNC) was defined according to the National Institute on Aging-Alzheimer’s Association (NIA-AA) ABC scoring system.^33^ Braak neurofibrillary tangle (NFT) stage (NIA-AA B score) was grouped into four ordered categories reflecting increasing severity (B0 = 0; B1 = I–II; B2 = III–IV; B3 = V–VI). Neocortical neuritic plaque density was assessed using the Consortium to Establish a Registry for ad (CERAD) score (NIA-AA C score).

### Genetic data processing

SNP data were obtained from the Alzheimer’s Disease Sequencing Project (ADSP)^34^^,^^35^ and the Alzheimer’s Disease Genomics Consortium (ADGC).^36^ For these two resources, we constructed two independent sample groups: participants in ADSP and NACC (hereafter referred to as “ADSP-NACC”) and participants in ADGC and NACC excluding those in ADSP-NACC (hereafter referred to as “ADGC-NACC”).

ADSP whole genome sequencing (WGS) data in release 5,^34^^,^^35^ were downloaded from the National Institute on Aging Genetics of Alzheimer’s Disease Data Storage Site (NIAGADS), which comprised 58 507 samples from 57 study cohorts.^34^^,^^35^ SNP assay data from ADRC1 to 17 batches, (which are a portion of Alzheimer’s Disease Genetics Consortium (ADGC) genotype data), were quality-controlled, removing variants with high missing rate (>5%) and rare variants (minor allele frequency <5%) and then imputed using the TOPMed Imputation Server (https://imputation.biodatacatalyst.nhlbi.nih.gov/)^37^ based on the Genome Reference Consortium Human Build 38 (GRCh38) reference panel. These genetic data were linked to clinical and neuropathological outcome data from the NACC dataset, as previously described.^38^

We then extracted SNP data for rs5848 located in *GRN* (coded as 0 = C/C, 1 = C/T, and 2 = T/T); rs1990622 located in *TMEM106B* (0 = G/G, 1 = G/A, and 2 = A/A); and rs744373 and rs6733839 located in *BIN1* (0 = A/A, 1 = A/G, and 2 = G/G in rs744373 and 0 = C/C, 1 = C/T, and 2 = T/T in rs6733839) from each data set. *APOE* ε4 dosage was derived from NACC UDS and coded as 0 = no ε4 allele, 1 = one ε4 allele, and 2 = two ε4 alleles.

We extracted phenotype-genotype data from participants with European ancestry. Individual genetic ancestry was inferred using principal component analysis (PCA) based on linkage disequilibrium (LD) pruned SNP assay data implemented in PLINK^39^ (—indep-pairwise 1500 50 0.2; window size = 1500 SNPs, sliding window increment = 50 SNPs, r^2^ threshold = 0.2), filtered by minor allele frequency (MAF ≥0.05) and genotype missingness (≤0.05), and excluding strand-ambiguous (A/T and C/G) variants. We performed PCA separately for the ADSP-NACC and ADGC-NACC datasets after merging each with genotype data from the 1000 Genomes Project Phase 3 (1000G),^40^^,^^41^ followed by uniform manifold approximation and projection (UMAP) for the first 20 principal components (PCs).^42^ A clustering-based approach was then applied to two-dimensional UMAP embeddings of study participants using k-means clustering with a pre-specified number of clusters (*k* = 15; random_state = 42) to capture fine-scale clustering patterns. Clusters were assigned to 1000G superpopulations based on proximity to the corresponding 1000G samples in the UMAP space, and participants assigned to the European reference cluster were extracted (Supplementary Figure 1, available at *Journal of Neuropathology & Experimental Neurology* online).^43^^,^^44^ To further adjust for residual European ancestry effects, PCA was rerun in PLINK using the “—pca” option, and a scree plot of eigenvalues was generated to determine the optimal number of PCs to include as covariates.

### Statistical analysis

**Figure 2:**
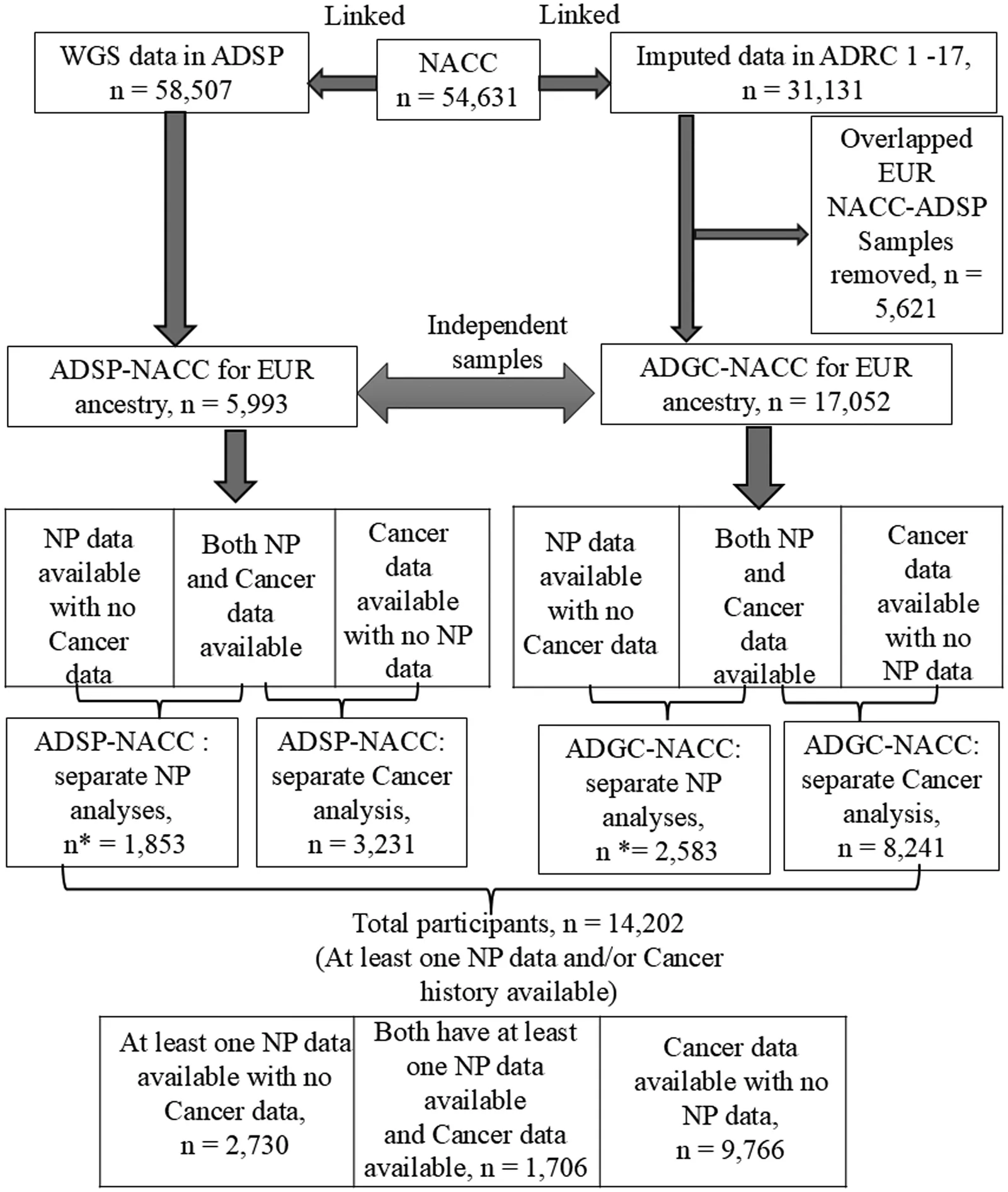
Study workflow and sample selection. n*: Number of samples with at least one neuropathology data. WGS: Whole Genome Sequencing, NP: Neuropathology, ADNC: NIA-AA Alzheimer’s disease neuropathologic change, ADSP: the Alzheimer’s Disease Sequencing Project, ADGC: Alzheimer’s Disease Genetics Consortium, NACC: National Alzheimer’s Coordinating Center, HS aging: Hippocampal sclerosis of Aging, including mesial temporal sclerosis.

Participants were included if NP data and/or cancer history were available, with missing data handled through an outcome-specific stratification, and analyses conducted within phenotype-specific subsets (Figure 2). Sensitivity analyses were restricted to individuals of European ancestry with complete data on both cancer status and hippocampal TDP-43 pathology for the rs5848 variant. Additionally, established Alzheimer disease risk variants of *TMEM106B, APOE ε4*, and *BIN1* were also evaluated for associations with cancer status to assess the specificity of the observed findings.

For binary outcomes, association testing was performed using the Scalable and Accurate Implementation of Generalized Mixed Model (SAIGE) with a binary outcome specification.^45^ SAIGE accounts for participant relatedness and addresses imbalance in the distribution of the modeled event outcomes through Firth’s Bias-Reduced Logistic Regression.^46^ For ordinal neuropathologic outcomes, we applied Proportional Odds Logistic Mixed Models (POLMM) using the Genome-wide Regression Analysis under Bias control (GRAB) framework,^47^ which extends cumulative logit models by incorporating random effects derived from a genetic relationship matrix to account for participant-relatedness and population structure.^47^^,^^48^ Ordinal outcomes included Braak NFT stages and CERAD neocortical neuritic plaque density.^49^^,^^50^ The proportional odds assumption was evaluated using the Brant test.^51^ Due to evidence that the proportional odds assumption was violated, the ADNC and CERAD neocortical neuritic plaque density outcomes were recategorized. Specifically, ADNC was dichotomized (ADNC: 0 = not ad, low ADNC, or intermediate ADNC vs 1 = high ADNC), and CERAD neocortical neuritic plaque density was trichotomized (0 = none, 1 = sparse, vs 2 = moderate or frequent). Each model was analyzed assuming an additive mode of inheritance and adjusted for sex, age at last visit for cancer history, age at death for neuropathological outcomes, and top 2-3 PCs as determined by inspection of scree plots of PCA eigenvalues derived from genotype data. Study-specific estimates of regression coefficients in ADSP-NACC and ADGC-NACC were meta-analyzed using METAL.^52^

## Results

The overall study workflow is summarized in Figure 2. Demographic and neuropathological characteristics of the ADSP-NACC and ADGC-NACC cohorts are presented in Table 1. The frequencies of rs5848 T-allele carriers across major cancer types are shown in Supplementary Table 2, available at *Journal of Neuropathology & Experimental Neurology* online. In the ADSP-NACC cohort, T-allele carrier frequencies ranged from 24.0% in lung cancer to 51.6% in breast cancer, whereas in the ADGC-NACC cohort, the range was from 41.4% in breast cancer to 55.4% in lung cancer.

**Table 1:** Demographic and neuropathological characteristics of ADGC and ADSP participants stratified by cancer status.

Characteristic, *n* (%) or mean ± SD	ADSP-NACC			ADGC-NACC		
	NP available*	No cancer	Cancer cases	NP available*	No cancer	Cancer cases
	(*n = *1853)	(*n = *2336)	(*n = *895)	(*n = *2583)	(*n = *5945)	(*n = *2296)
Age at death	79.0 ± 10.8	78.5 ± 11.1	84.0 ± 8.6	76.9 ± 10.0	84.0 ± 10.6	85.5 ± 8.8
Hippocampal sclerosis of aging†	216 (13.0)	84(15.9)	19 (11.4)	297 (14.7)	100 (14.6)	39 (13.5)
TDP-43 pathology						
Amygdala	252 (15.2)	158 (37.4)	48 (36.1)	331 (16.3)	185 (33.0)	79 (31.7)
Hippocampus	222 (13.4)	124 (27.7)	37 (26.2)	297 (14.7)	157 (27.2)	59 (23.0)
Neocortex	39 (2.4)	25 (6.3)	5 (3.9)	70 (3.5)	34 (6.5)	12 (5.1)
Braak NFT stage						
0	15 (0.8)	1 (0.2)	2 (1.2)	97 (3.9)	22 (3.1)	6 (2.1)
I-II	196 (10.6)	58 (10.8)	32 (18.8)	485 (19.7)	131 (18.7)	60 (20.8)
III-IV	377 (20.4)	91 (16.9)	39 (22.9)	655 (26.6)	170 (24.2)	89 (30.8)
V-VI	1261 (68.2)	389 (72.2)	97 (57.1)	1229 (49.8)	379 (54.0)	134 (46.4)
CERAD neuritic plaque score						
No	534 (21.6)	70 (13.0)	38 (22.4)	224 (12.1)	152(21.6)	72 (24.9)
Sparse	352 (14.2)	56 (10.4)	25 (14.7)	191 (10.3)	77(10.9)	35 (12.1)
Moderate + Severe	1588 (64.2)	413(76.6)	107 (62.9)	1436 (77.6)	475 (67.5)	182 (63.0)
ADNC						
High ADNC	738 (44.5)	360 (67.0)	80 (47.1)	690 (34.0)	335 (48.1)	107 (37.2)

* Participants with available neuropathologic assessments used for genetic analysis, with or without cancer history. ^†^: Hippocampal sclerosis of Aging, including mesial temporal sclerosis, NP: Neuropathology, ADNC: NIA-AA Alzheimer’s disease neuropathologic change, ADSP: the Alzheimer’s Disease Sequencing Project, ADGC: Alzheimer’s Disease Genetics Consortium, NACC: National Alzheimer’s Coordinating Center, SD: Standard Deviation, NFT: Neurofibrillary Tangles, CERAD: Consortium to Establish a Registry for Alzheimer’s Disease.

**Figure 3:**
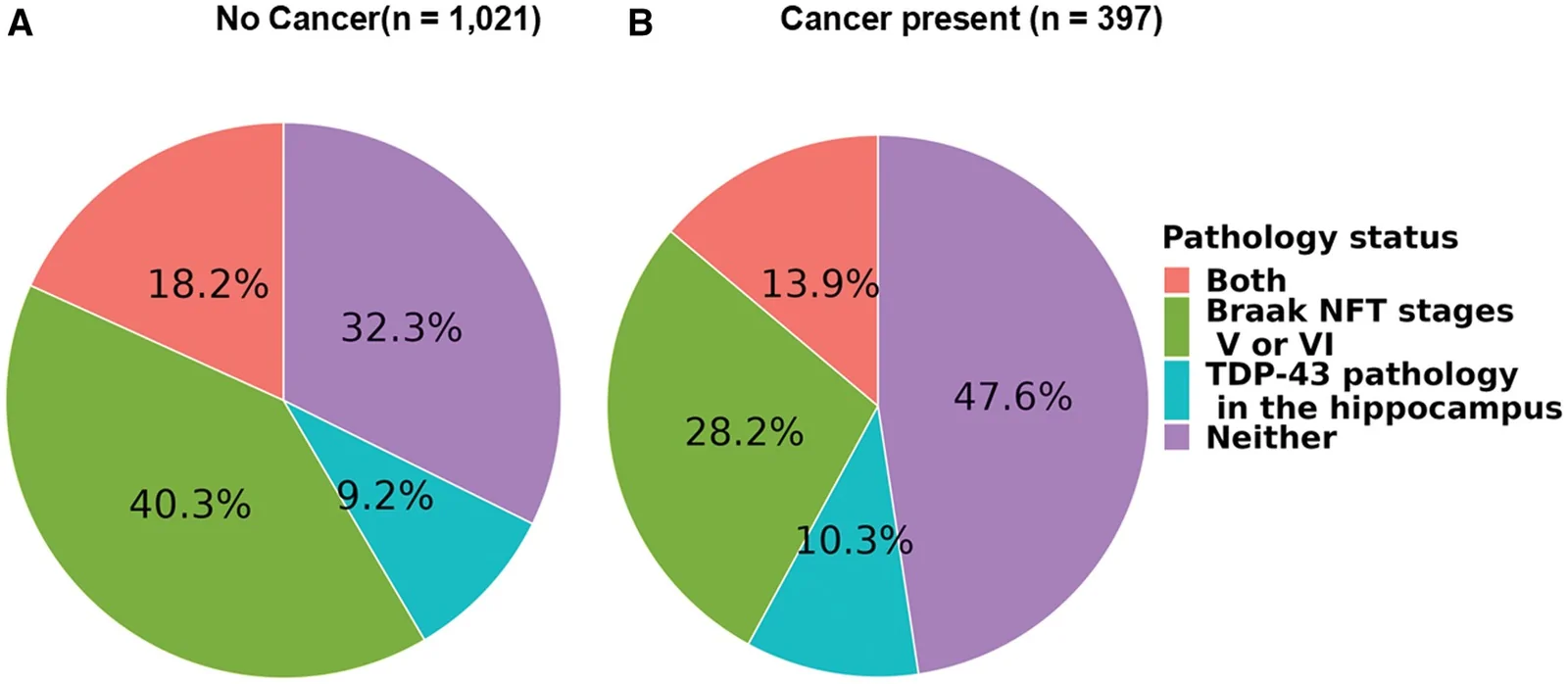
(A, B) Distribution of Braak NFT stages V or VI and TDP-43 pathology in hippocampus among pooled ADGC and ADSP participants stratified by cancer history. Analyses were restricted to participants with complete data on of Braak NFT stages V or VI and TDP-43 pathology in the hippocampus, and cancer status.

Among participants without a reported history of cancer who had complete data on both Braak NFT stages and TDP-43 pathology in the hippocampus (Figure 3), 40.3% had Braak NFT stages V or VI, 9.2% had TDP-43 pathology in the hippocampus, 18.2% had both pathologies, and 32.3% had neither (Figure 3A). In contrast, among participants with cancer history, a larger proportion had neither pathology (47.6%), while 28.2% had Braak NFT stages V or VI, 10.3% had TDP43 pathology in the hippocampus, and 13.9% had both pathologies (Figure 3B).

**Figure 4:**
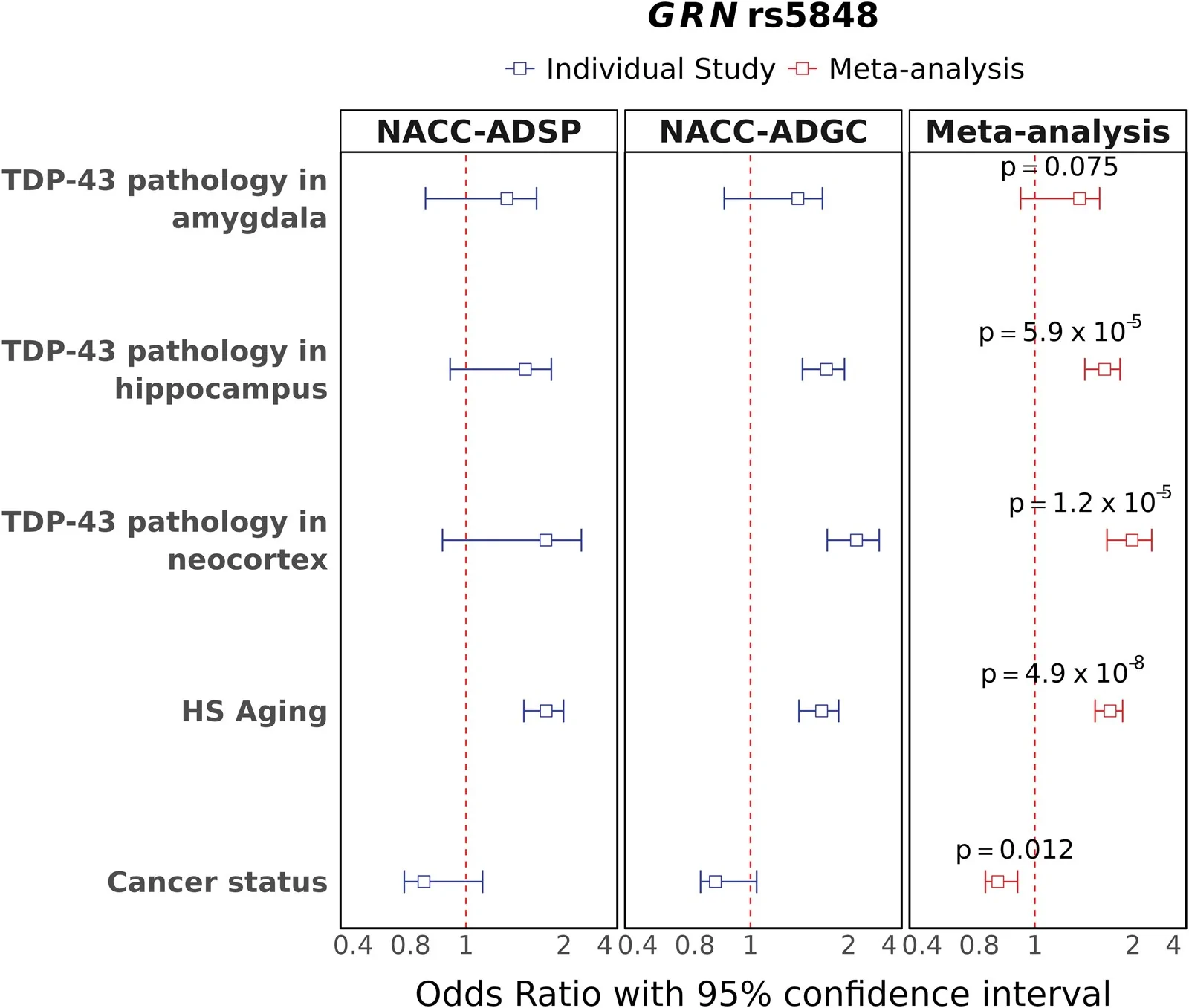
Forest plots for the association of the T allele rs5848 in *GRN* with TDP-43 pathology, HS Aging and cancer status across cohorts. The red dashed vertical line corresponds to the null hypothesis of no association (odds ratio = 1). Genetic association analyses were performed using SAIGE logistic mixed models, adjusting for ancestry principal components, sex, and age. Age at death was used as an age covariate for neuropathologic outcomes in genetic analyses, while age at last clinical assessment was used for cancer outcomes, with cancer defined as having been reported at least once during follow-up. ADSP: the Alzheimer’s Disease Sequencing Project, ADGC: Alzheimer’s Disease Genetics Consortium, NACC: National Alzheimer’s Coordinating Center, HS aging: Hippocampal sclerosis of Aging, including mesial temporal sclerosis.

In the meta-analysis across the ADSP-NACC and ADGC-NACC cohorts, the T allele of rs5848 was associated with increased odds of TDP-43 pathology in hippocampus (OR = 1.42, 95% CI = 1.20-1.69, *p = *5.9 × 10^−5^) and neocortex (OR = 1.97, 95% CI = 1.46-2.68, *p = *1.2 × 10^−5^), but not in amygdala (OR = 1.16, CI = 0.99-1.35, *p = *0.075). The association with HS-Aging was significant in both cohorts and in the meta-analysis (OR = 1.50, CI = 1.30-1.74, *p = *4.9 × 10^−8^). In contrast, a significant protective association with cancer history was observed in the meta-analysis (OR = 0.91, 95% CI = 0.84-0.98, *p = *0.012) (Figure 4).

**Figure 5:**
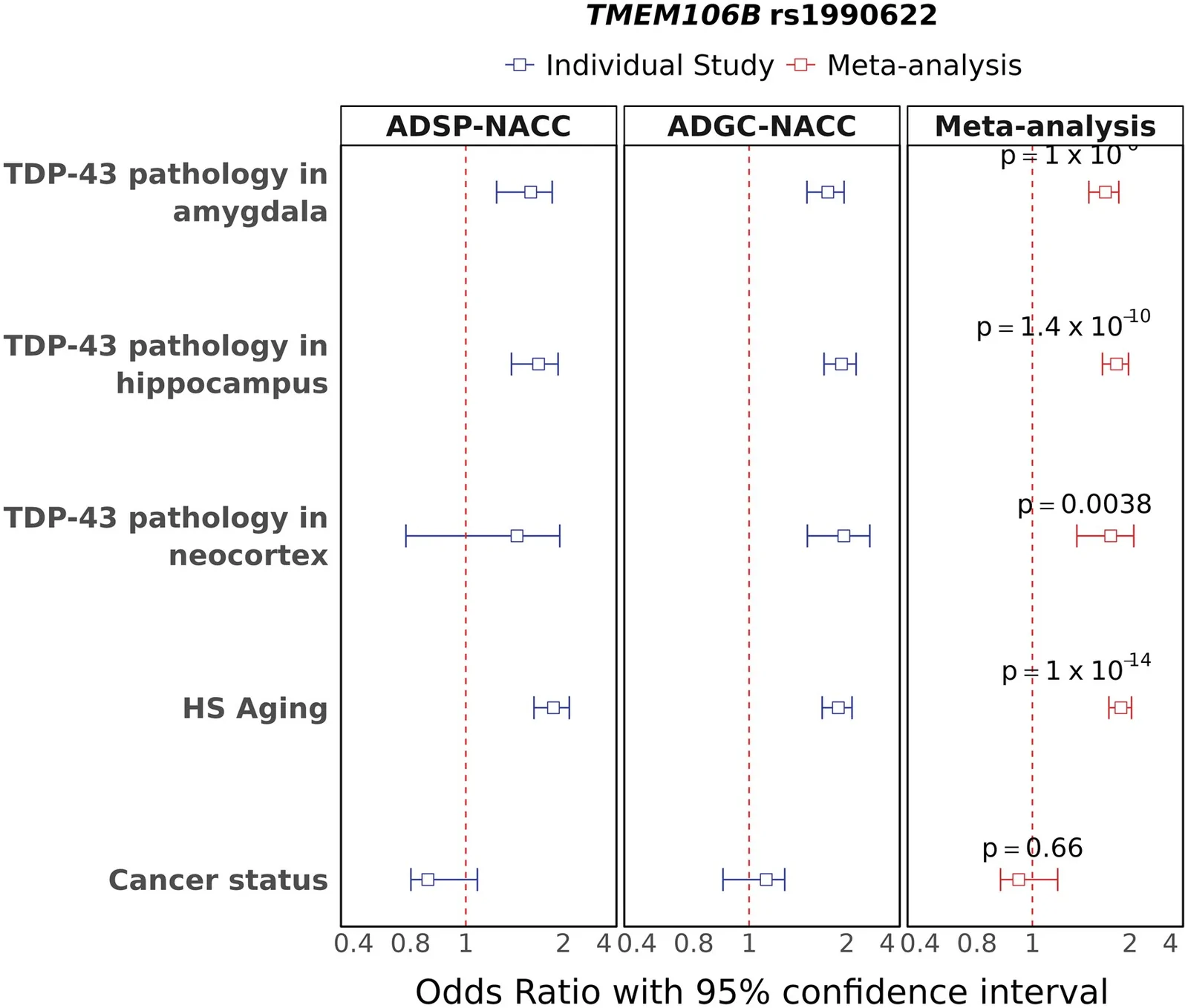
Forest plots of the association between the *TMEM106B* rs1990622 A allele and TDP-43 pathology, HS Aging, and cancer status across cohorts. The red dashed vertical line corresponds to the null hypothesis of no association (odds ratio = 1). Genetic association analyses were performed using Scalable and Accurate Implementation of Generalized Linear Mixed Models (SAIGE) logistic mixed models, adjusting for ancestry principal components, sex, and age. Age at death was used as an age covariate for neuropathologic outcomes in genetic analyses, while age at last clinical assessment was used for cancer outcomes, with cancer defined as having been reported at least once during follow-up. ADSP: the Alzheimer’s Disease Sequencing Project, ADGC: Alzheimer’s Disease Genetics Consortium, NACC: National Alzheimer’s Coordinating Center, HS aging: Hippocampal sclerosis of Aging, including mesial temporal sclerosis.

In the parallel analysis of the *TMEM106B* rs1990622, a well-established TDP-43–related SNP, the A allele was strongly associated with an increased odds of TDP-43 pathology in amygdala and hippocampus and HS-Aging in both the ADSP-NACC and ADGC-NACC cohorts. The meta-analysis demonstrated robust pooled effects on HS-Aging (OR = 1.77, 95% CI =1.53-2.04, *p = *1.0 × 10^−14^) and on TDP-43 pathology in amygdala and hippocampus (OR point estimate range was 1.47 to 1.67 and both p-values were < 1 × 10^−6^), indicating that the A allele confers increased susceptibility to HS-Aging as well as TDP-43–related neuropathology in amygdala and hippocampus. In contrast, the meta-analysis showed no evidence of an association with cancer status (OR = 0.99, 95% CI = 0.93-1.05, *p = *0.66) (Figure 5).

**Figure 6:**
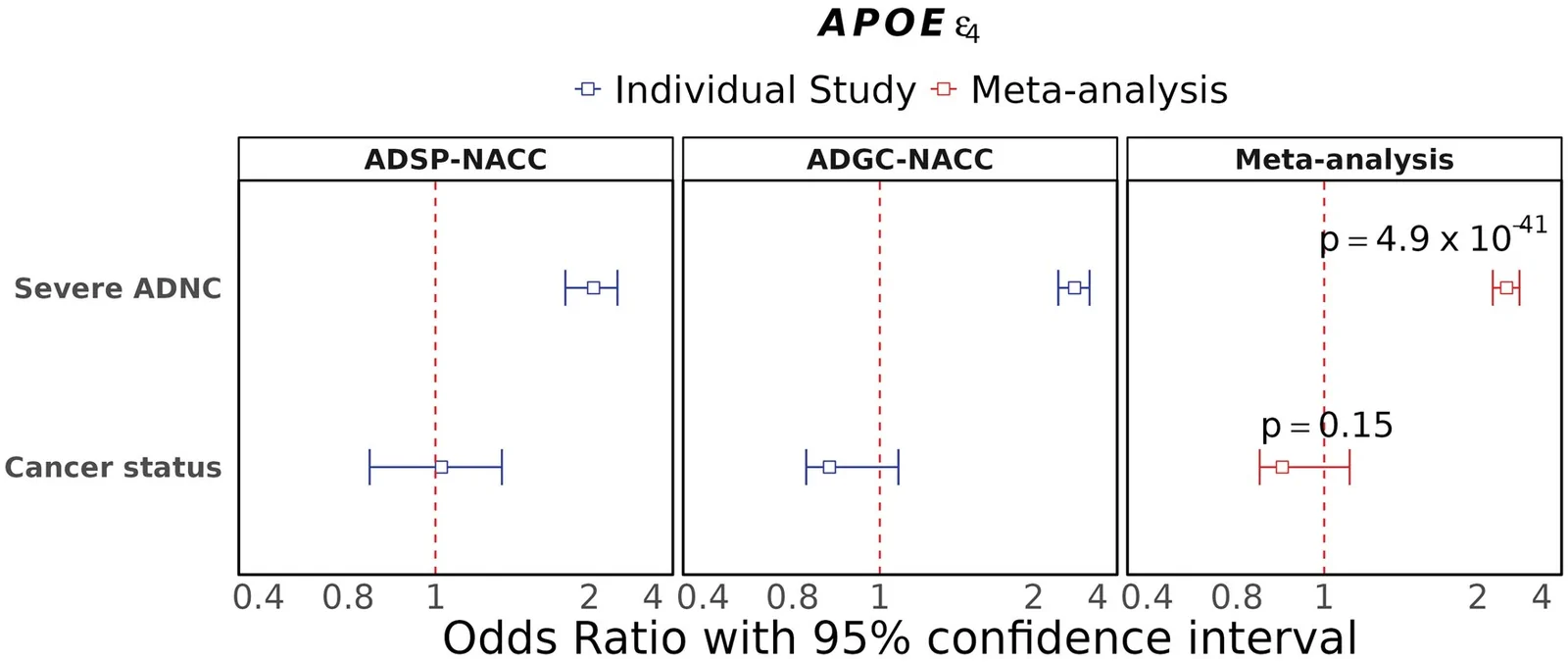
Forest plots showing the association of *APOE* ε4 with Alzheimer disease–related neuropathologic changes and reported cancer history across two cohorts (ADSP-NACC and ADGC-NACC), followed by fixed-effect meta-analysis. ADNC scores and cancer status were analyzed as binary traits. The red dashed vertical line corresponds to the null hypothesis of no association (odds ratio = 1). Genetic association analyses were performed using Scalable and Accurate Implementation of Generalized Linear Mixed Models (SAIGE) logistic mixed models, adjusting for ancestry principal components, sex, and age. Age at death was used as an age covariate for neuropathologic outcomes in genetic analyses, while age at last clinical assessment was used for cancer outcomes, with cancer defined as having been reported at least once during follow-up. ADNC: NIA-AA Alzheimer’s disease neuropathologic change, ADSP: the Alzheimer’s Disease Sequencing Project, ADGC: Alzheimer’s Disease Genetics Consortium, NACC: National Alzheimer’s Coordinating Center.

**Figure 7:**
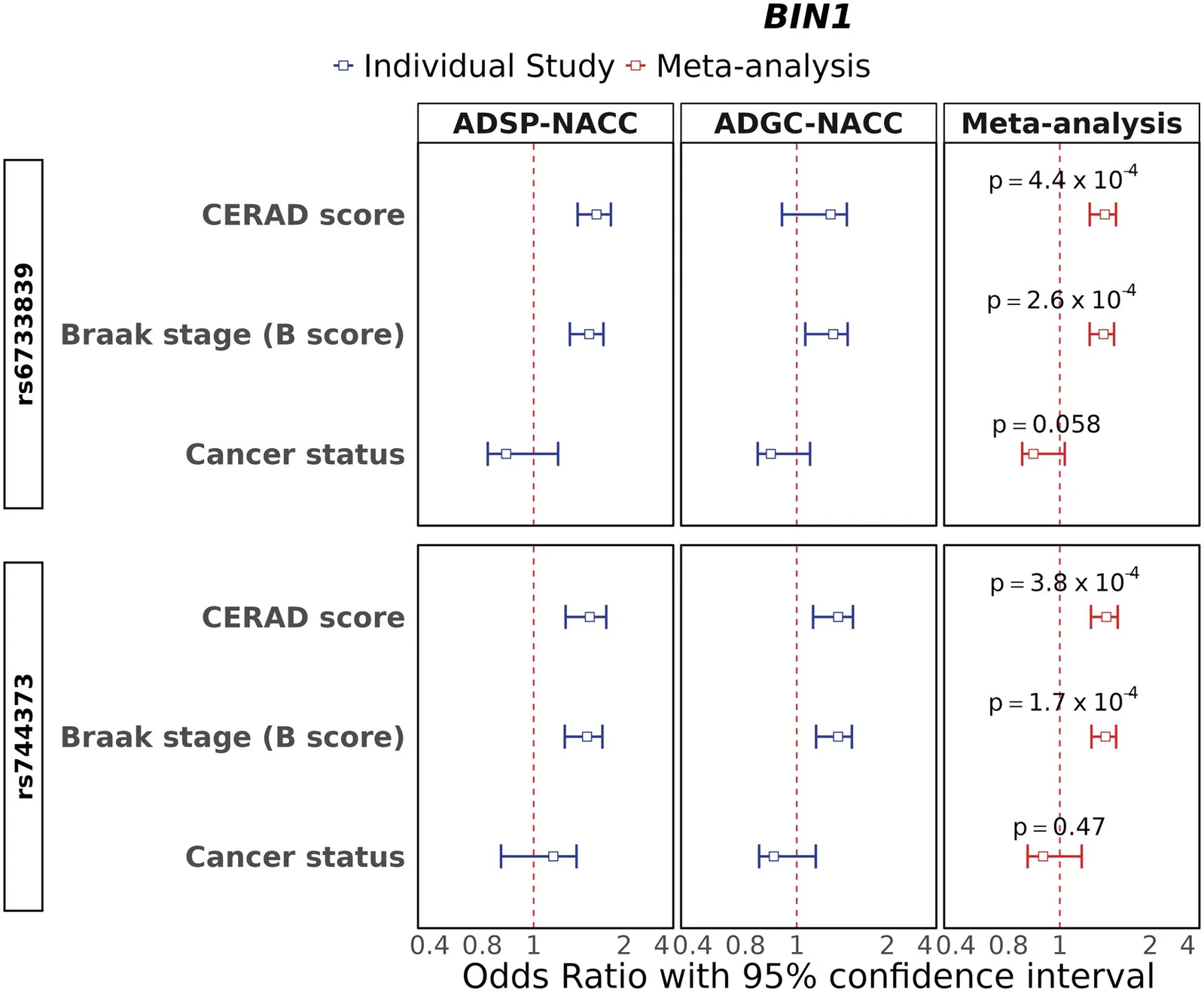
Forest plots showing the association of two *BIN1* variants—rs6733839 (G allele), rs744373(T allele) with Alzheimer’s disease–related neuropathologic changes and reported cancer history across two cohorts (ADSP and ADGC), followed by fixed-effect meta-analysis. Neuropathologic outcomes (CERAD score, Braak B score) were analyzed as ordinal traits using proportional odds logistic mixed models, whereas cancer status was analyzed as a binary outcome using Scalable and Accurate Implementation of Generalized Linear Mixed Models (SAIGE) logistic mixed models. All models were adjusted for sex, principal components of ancestry, and age. Age at death was used as an age covariate for neuropathologic outcomes in genetic analyses, while age at last clinical assessment was used for cancer outcomes, with cancer defined as having been reported at least once during follow-up. ADSP: the Alzheimer’s Disease Sequencing Project, ADGC: Alzheimer’s Disease Genetics Consortium, NACC: National Alzheimer’s Coordinating Center, CERAD score: Consortium to Establish a Registry for Alzheimer’s Disease neuritic plaque score, Braak stage: Braak neurofibrillary tangle stage.

We also assessed whether similar protective associations of other ad-related SNPs (analogous to those observed for rs5848) could be detected. As shown in Figure 6, *APOE* ε4 was not associated with cancer in either cohort or in the meta-analysis (OR = 0.95, 95% CI 0.89-1.02; *p = *0.15). Additionally, two ad-associated variants, rs744373 (G allele) and rs6733839 (T allele), located in *BIN1* had robust significant associations with neocortical neuritic plaques and Braak NFT stage but not with cancer diagnosis (Figure 7). All detailed cohort-specific and meta-analyzed results are provided in Supplementary Table 3, available at *Journal of Neuropathology & Experimental Neurology* online. Analyses were subsequently focused on two major cancer types, breast and prostate cancers, to assess associations of the rs5848 T allele (with the implicit caveat of smaller sample sizes). Although a significant protective association of T allele with breast cancer was observed in the ADGC-NACC cohort, this was not replicated in the ADSP-NACC cohort and meta-analysis (Supplementary Table 4, available at *Journal of Neuropathology & Experimental Neurology* online).

In sensitivity analyses restricted to the subsample of included participants with available data on both history of cancer and TDP-43 pathology in hippocampus (*n = *1,423; demographic and neuropathological characteristics shown in Supplementary Table 5, available at *Journal of Neuropathology & Experimental Neurology* online), the rs5848 T allele was not associated with cancer history in the meta-analysis (OR = 0.97, 95% CI = 0.80-1.17), whereas its association with TDP-43 pathology in hippocampus remained significant (OR = 1.52, 95% CI 1.25-1.84). We additionally evaluated the interaction effect between cancer history and rs5848 T allele on LATE-NC stages (0 to 3) using an ordinal regression analysis, showing that rs5848 T allele did not modify the relationship between cancer history and LATE-NC severity (Supplementary Table 6, available at *Journal of Neuropathology & Experimental Neurology* online).

## Discussion

Analyzing data from two cohorts, we found, as expected, that the T allele of rs5848 in *GRN* was associated with increased burden of LATE-NC and HS-Aging pathologies. In contrast, the same allele was associated with lower odds of documented cancer history. The findings of a negative association between the *GRN* dementia-related risk allele with cancer history were different than for other dementia-related risk alleles. Specifically, established *TMEM106B, APOE*, and *BIN1* risk alleles had no significant associations with cancer history in the same sample.

Although the T allele of rs5848 has been extensively studied as a risk factor for HS-Aging, LATE-NC, and other TDP-43-related proteinopathies in older adults, its relationship with cancer has not previously been examined, despite long-standing evidence that PGRN has potential roles in tumor biology.^13^ One exception to this was the work of Rosas et al that showed in a cohort of 753 Parkinson disease patients, the rs5848 T allele was associated with lower cancer likelihood.^27^ Otherwise, our findings provide novel evidence that rs5848 T allele confers a protective effect against cancer in later life.

Mechanistically, a single genetic variant with opposite impacts across different clinical syndromes is biologically plausible. Previous studies showed that rs5848, located in the 3'-UTR of *GRN*, was functional. The T allele reduced *GRN* translation (ie, rs5848 acts as an expression quantitative trait locus [eQTL]), leading to lower PGRN levels in blood^24^ and was linked to increased expression of inflammatory mediators central nervous system (CNS) such as *AXL* and *CLU*, reflecting PGRN’s role in regulating inflammation and lysosomal function.^11^^,^^53–55^ Remarkably, rs5848 has been associated a diverse set of conditions linked to inflammation such as Parkinson disease, ad, and multiple sclerosis.^22^^,^^56^^,^^57^ Given that reduced *GRN*/PGRN levels, particularly associated with the rs5848 T allele, have been consistently linked to neurodegeneration (Supplementary Table 7, available at *Journal of Neuropathology & Experimental Neurology* online),^14^^,^^16^^,^^17^^,^^20^^,^^22^^,^^24^^,^^25^^,^^53^^,^^55–65^ PGRN deficiency appears to promote neuroinflammation and TDP-43 pathology. By contrast, increased *GRN*/PGRN expression may have the opposite consequence in oncogenesis. Prior studies reported that *GRN*/PGRN plays an important role across multiple cancers, where its expression/overexpression promotes tumor cell growth, migration, and invasivity (Supplementary Table 8, available at *Journal of Neuropathology & Experimental Neurology* online).^13^^,^^27^^,^^66–83^ Increased PGRN expression in blood and/or in CNS tissue may attenuate inflammation while promoting tumor proliferation. Together, these observations support a mechanistic model in which *GRN*/PGRN-mediated regulation of inflammatory, lysosomal, and mitogenic pathways creates a biological tradeoff, offering a plausible mechanistic basis for the opposing associations between rs5848 and dementia versus cancer-related outcomes.

Our interaction analysis indicated that rs5848 does not function as an effect modifier in the association between cancer and TDP-43 pathology suggesting that the presence of the rs5848 T allele does not alter the strength or direction of this association. Instead, these results support a model in which the rs5848 T allele independently influences the risk for both disease domains. This in turn implies that the mechanisms linking rs5848 to cancer and to TDP-43 pathology may operate through concurrent but not necessarily identical biological pathways, providing a remarkable example of an impactful pleiotropic mechanism.

These results have clinical implications. Decreased blood and organ PGRN levels (as with the rs5848 T allele, and also in persons with homozygous or heterozygous deletions of the functional *GRN* gene), are associated with several human diseases, including FTLD-TDP, LATE-NC, and neuronal ceroid lipofuscinosis.^62–65^^,^^84–88^ The possibility of augmenting *GRN*/PGRN expression in vivo has catalyzed exciting work aimed at clinical therapies that are designed to augment PGRN levels in blood and/or brain.^58–61^ However, among gliomas and other subtypes of cancer, PGRN has been shown to stimulate cell growth and malignancy.^66–68^^,^^89^ The present study underscores that efforts to increase PGRN expression should be undertaken with caution due to the potential increased risk of neoplasia.

We also note that our results shed light on the reciprocal relationship between LATE-NC/HS-Aging and cancer but not that between ADNC and cancer. However, the distributions of autopsy-confirmed dementia-associated pathologies in individuals with and without cancer diagnoses provided (admittedly limited) support for the hypothesis that ADNC and cancer are indeed reciprocally associated (Figure 3). Among the three specific alleles that were queried for associations between ADNC and cancer (2 *BIN1* SNPs, and *APOE* alleles), one of the *BIN1* SNPs (rs6733839) had a dementia risk allele with marginal trend (*p < *0.06) in meta-analyzed data for negative association with cancer risk (Figure 7). There are also a number of prior published studies that have indeed indicated a potential role for *BIN1* in neoplasia transformation or progression,^90–92^ possibly via inflammatory mechanisms, and a cancer-protective role for the dementia-risk associated allele(s) of *BIN1* remains an area for future studies.

Our study has some limitations. Firstly, cancer history was ascertained using the NACC UDS version 3.0 Form D2, in which cancer is defined as “cancer present in the last 12 months (excluding non-melanoma skin cancer), primary or metastatic.” Because this variable was introduced in March 2015, individuals who were treated for and survived cancer prior to March 2014 may have been coded as cancer-free despite having a true prior cancer history.^30^ Moreover, the 12-month time restriction was specified in the coding guidebook but not explicitly included on the data collection form, so there is potential for heterogeneity in data collection across contributing ADRCs, where some may have considered cancers from earlier in life while others did not. Therefore, we consider this a measure of later life cancer history that likely has higher sensitivity than specificity. However, misclassification probability is almost certainly nondifferential with respect to genetic profiles, since genetic profiles would have been unknown to both participants and clinicians at the time of data collection. As such, we anticipate that our observed results for associations with later life cancer may be biased toward the null. Our study design also lacked direct histopathological correlations between tumor tissue subtypes and dementia-related neuropathology. Moreover, cancer type–specific analyses were under-powered statistically, highlighting the need for larger studies to clarify potential cancer subtype-specific effects. In principle, the molecular “fingerprinting” of cancer subtypes should go beyond histological classification. Likewise, on the other side of the associative comparison, fully quantitative neuropathological measures (eg, TDP-43–immunopositive area or cell counts), along with more detailed qualitative assessment of TDP-43 pathology, will be also required in future investigations.

Our analyses were also limited to participants with European ancestry. This study design feature is commonly employed to minimize genotype variation due to ethnoracial genetic architectural differences. However, this consideration is doubly relevant because we previously described that the rs5848 risk allele has far higher population frequency in persons of African ancestry than in those with European ancestry.^23^ Theoretically this may indicate a genetic factor associated with lower cancer risk and higher dementia risk in person of African ancestry. Again, we emphasize that larger and more diverse cohorts are required to study these research questions in a more generalizable manner.

We also acknowledge the possibility of survival bias and recruitment biases. These are known features of the dementia-associated study cohorts that were used in the present study.^32^ As such, these observations are preliminary, and future analyses incorporating formal survival modelling and longitudinal follow-up will be necessary to determine whether age-related, socioeconomic factors, and other cohort-specific selection effects contribute to the phenomena that were described in the current study.

Despite these limitations, the proportion of participants with a documented later life cancer history in the present dataset (∼28%) is within a plausible range when compared to population-based estimates of lifetime cancer risk (∼39%) and cancer mortality (∼17%).^93^ Considered together, these observations suggest modest under-recruitment, and/or under-reporting, of individuals with later life cancer rather than severe misclassification. Future studies using datasets with more comprehensive lifetime cancer ascertainment may help further clarify these associations.

In summary, our study provides evidence that the PGRN-lowering rs5848 T allele is associated with reduced cancer burden while simultaneously conferring increased susceptibility to LATE-NC. The robustness of the rs5848 associations across multiple cohorts, together with the absence of comparable signals for *TMEM106B, APOE* ε4 or *BIN1* variants in relation to cancer, underscores at least partial specificity of this genetic association. Although survival bias cannot be fully excluded, the opposing associations suggest that rs5848 may differentially influence two common aging-related phenotypes, each with large public health impact. Future studies across larger, diverse populations, with more comprehensive data gathered including detailed cancer phenotyping, will be essential to clarify the *GRN*-mediated disease risk trade-off and to help inform how *GRN*/PGRN-related pathways could be targeted for therapeutic development.

## Supplementary Material

nlag051_Supplementary_Data

## Data Availability

All data, and novel reagents, used in this article will be made available to the research community in a reasonable timeframe.

## ADGC

The Alzheimer’s Disease Genetics Consortium (ADGC) is funded by a grant from the National Institute on Aging (PI, Gerard D. Schellenberg; UO1AG032984).

The National Institutes of Health, National Institute on Aging (NIH-NIA) supported this work through the following grants: ADGC, U01 AG032984, RC2 AG036528; Samples from the National Cell Repository for Alzheimer’s Disease (NCRAD), which receives government support under a cooperative agreement grant (U24 AG21886) awarded by the National Institute on Aging (NIA), were used in this study.

Data for this study were prepared, archived, and distributed by the National Institute on Aging Alzheimer’s Disease Data Storage Site (NIAGADS) at the University of Pennsylvania (U24-AG041689-01); NACC, U01 AG016976; NIA LOAD, U24 AG026395, R01AG041797; Banner Sun Health Research Institute P30 AG019610; Boston University, P30 AG013846, U01 AG10483, R01 CA129769, R01 MH080295, R01 AG017173, R01 AG025259, R01AG33193; Columbia University, P50 AG008702, R37 AG015473; Duke University, P30 AG028377, AG05128; Emory University, AG025688; Group Health Research Institute, UO1 AG006781, UO1 HG004610, UO1 HG006375; Indiana University, P30 AG10133; Johns Hopkins University, P50 AG005146, R01 AG020688; Massachusetts General Hospital, P50 AG005134; Mayo Clinic, P50 AG016574; Mount Sinai School of Medicine, P50 AG005138, P01 AG002219; New York University, P30 AG08051, UL1 RR029893, 5R01AG012101, 5R01AG022374, 5R01AG013616, 1RC2AG036502, 1R01AG035137; Northwestern University, P30 AG013854; Oregon Health & Science University, P30 AG008017, R01 AG026916; Rush University, P30 AG010161, R01 AG019085, R01 AG15819, R01 AG17917, R01 AG30146; TGen, R01 NS059873; University of Alabama at Birmingham, P50 AG016582; University of Arizona, R01 AG031581; University of California, Davis, P30 AG010129; University of California, Irvine, P50 AG016573; University of California, Los Angeles, P50 AG016570; University of California, San Diego, P50 AG005131; University of California, San Francisco, P50 AG023501, P01 AG019724; University of Kentucky, P30 AG028383, AG05144; University of Michigan, P50 AG008671; University of Pennsylvania, P30 AG010124; University of Pittsburgh, P50 AG005133, AG030653, AG041718, AG07562, AG02365; University of Southern California, P50 AG005142; University of Texas Southwestern, P30 AG012300; University of Miami, R01 AG027944, AG010491, AG027944, AG021547, AG019757; University of Washington, P50 AG005136; University of Wisconsin, P50 AG033514; Vanderbilt University, R01 AG019085; and Washington University, P50 AG005681, P01 AG03991.

The Kathleen Price Bryan Brain Bank at Duke University Medical Center is funded by NINDS grant # NS39764, NIMH MH60451 and by Glaxo Smith Kline. Genotyping of the TGEN2 cohort was supported by Kronos Science. The TGen series was also funded by NIA grant AG041232 to AJM and MJH, The Banner Alzheimer’s Foundation, The Johnnie B. Byrd Sr.

Alzheimer’s Institute, the Medical Research Council, and the state of Arizona and also includes samples from the following sites: Newcastle Brain Tissue Resource (funding via the Medical Research Council, local NHS trusts and Newcastle University), MRC London Brain Bank for Neurodegenerative Diseases (funding via the Medical Research Council),South West Dementia Brain Bank (funding via numerous sources including the Higher Education Funding Council for England (HEFCE), Alzheimer’s Research Trust (ART), BRACE as well as North Bristol NHS Trust Research and Innovation Department and DeNDRoN), The Netherlands Brain Bank (funding via numerous sources including Stichting MS Research, Brain Net Europe, Hersenstichting Nederland Breinbrekend Werk, International Parkinson Fonds, Internationale Stiching Alzheimer Onderzoek), Institut de Neuropatologia, Servei Anatomia Patologica, Universitat de Barcelona.

ADNI data collection and sharing was funded by the National Institutes of Health Grant U01 AG024904 and Department of Defense award number W81XWH-12-2-0012. ADNI is funded by the National Institute on Aging, the National Institute of Biomedical Imaging and Bioengineering, and through generous contributions from the following: AbbVie, Alzheimer’s Association; Alzheimer’s Drug Discovery Foundation; Araclon Biotech; BioClinica, Inc.; Biogen; Bristol-Myers Squibb Company; CereSpir, Inc.; Eisai Inc.; Elan Pharmaceuticals, Inc.; Eli Lilly and Company; EuroImmun; F. Hoffmann-La Roche Ltd and its affiliated company Genentech, Inc.; Fujirebio; GE Healthcare; IXICO Ltd; Janssen Alzheimer Immunotherapy Research & Development, LLC.; Johnson & Johnson Pharmaceutical Research & Development LLC.; Lumosity; Lundbeck; Merck & Co., Inc.; Meso Scale Diagnostics, LLC.; NeuroRx Research; Neurotrack Technologies; Novartis Pharmaceuticals Corporation; Pfizer Inc.; Piramal Imaging; Servier; Takeda Pharmaceutical Company; and Transition Therapeutics.

The Canadian Institutes of Health Research is providing funds to support ADNI clinical sites in Canada. Private sector contributions are facilitated by the Foundation for the National Institutes of Health (www.fnih.org).

The grantee organization is the Northern California Institute for Research and Education, and the study is coordinated by the Alzheimer’s Disease Cooperative Study at the University of California, San Diego. ADNI data are disseminated by the Laboratory for Neuro Imaging at the University of Southern California. We thank Drs. D. Stephen Snyder and Marilyn Miller from NIA who are ex-officio ADGC members.

Support was also from the Alzheimer’s Association (LAF, IIRG-08 to 89720; MP-V, IIRG-05 to 14147) and the US Department of Veterans Affairs Administration, Office of Research and Development, Biomedical Laboratory Research Program. P.S.G.-H. is supported by Wellcome Trust, Howard Hughes Medical Institute, and the Canadian Institute of Health Research.

## ADSP

The Alzheimer’s Disease Sequencing Project (ADSP) is comprised of two Alzheimer’s Disease (ad) genetics consortia and three National Human Genome Research Institute (NHGRI) funded Large Scale Sequencing and Analysis Centers (LSAC). The two ad genetics consortia are the Alzheimer’s Disease Genetics Consortium (ADGC) funded by NIA (U01 AG032984), and the Cohorts for Heart and Aging Research in Genomic Epidemiology (CHARGE) funded by NIA (R01 AG033193), the National Heart, Lung, and Blood Institute (NHLBI), other National Institute of Health (NIH) institutes and other foreign governmental and non-governmental organizations. The Discovery Phase analysis of sequence data is supported through UF1AG047133 (to Drs. Schellenberg, Farrer, Pericak-Vance, Mayeux, and Haines); U01AG049505 to Dr Seshadri; U01AG049506 to Dr Boerwinkle; U01AG049507 to Dr Wijsman; and U01AG049508 to Dr Goate and the Discovery Extension Phase analysis is supported through U01AG052411 to Dr Goate, U01AG052410 to Dr Pericak-Vance and U01 AG052409 to Drs. Seshadri and Fornage.

Sequencing for the Follow Up Study (FUS) is supported through U01AG057659 (to Drs. PericakVance, Mayeux, and Vardarajan) and U01AG062943 (to Drs. Pericak-Vance and Mayeux). Data generation and harmonization in the Follow-up Phase is supported by U54AG052427 (to Drs. Schellenberg and Wang). The FUS Phase analysis of sequence data is supported through U01AG058589 (to Drs. Destefano, Boerwinkle, De Jager, Fornage, Seshadri, and Wijsman), U01AG058654 (to Drs. Haines, Bush, Farrer, Martin, and Pericak-Vance), U01AG058635 (to Dr Goate), RF1AG058066 (to Drs. Haines, Pericak-Vance, and Scott), RF1AG057519 (to Drs. Farrer and Jun), R01AG048927 (to Dr Farrer), and RF1AG054074 (to Drs. Pericak-Vance and Beecham).

The ADGC cohorts include: Adult Changes in Thought (ACT) (U01 AG006781, U19 AG066567), the Alzheimer’s Disease Research Centers (ADRC) (P30 AG062429, P30 AG066468, P30 AG062421, P30 AG066509, P30 AG066514, P30 AG066530, P30 AG066507, P30 AG066444, P30 AG066518, P30 AG066512, P30 AG066462, P30 AG072979, P30 AG072972, P30 AG072976, P30 AG072975, P30 AG072978, P30 AG072977, P30 AG066519, P30 AG062677, P30 AG079280, P30 AG062422, P30 AG066511, P30 AG072946, P30 AG062715, P30 AG072973, P30 AG066506, P30 AG066508, P30 AG066515, P30 AG072947, P30 AG072931, P30 AG066546, P20 AG068024, P20 AG068053, P20 AG068077, P20 AG068082, P30 AG072958, P30 AG072959), the Chicago Health and Aging Project (CHAP) (R01 AG11101, RC4 AG039085, K23 AG030944), Indiana Memory and Aging Study (IMAS) (R01 AG019771), Indianapolis Ibadan (R01 AG009956, P30 AG010133), the Memory and Aging Project (MAP) (R01 AG17917), Mayo Clinic (MAYO) (R01 AG032990, U01 AG046139, R01 NS080820, RF1 AG051504, P50 AG016574), Mayo Parkinson’s Disease controls (NS039764, NS071674, 5RC2HG005605), University of Miami (R01 AG027944, R01 AG028786, R01 AG019085, IIRG09133827, A2011048), the Multi-Institutional Research in Alzheimer’s Genetic Epidemiology Study (MIRAGE) (R01 AG09029, R01 AG025259), the National Centralized Repository for Alzheimer’s Disease and Related Dementias (NCRAD) (U24 AG021886), the National Institute on Aging Late Onset Alzheimer’s Disease Family Study (NIA- LOAD) (U24 AG056270), the Religious Orders Study (ROS) (P30 AG10161, R01 AG15819), the Texas Alzheimer’s Research and Care Consortium (TARCC) (funded by the Darrell K Royal Texas Alzheimer’s Initiative), Vanderbilt University/Case Western Reserve University (VAN/CWRU) (R01 AG019757, R01 AG021547, R01 AG027944, R01 AG028786, P01 NS026630, and Alzheimer’s Association), the Washington Heights-Inwood Columbia Aging Project (WHICAP) (RF1 AG054023), the University of Washington Families (VA Research Merit Grant, NIA: P50AG005136, R01AG041797, NINDS: R01NS069719), the Columbia University Hispanic Estudio Familiar de Influencia Genetica de Alzheimer (EFIGA) (RF1 AG015473), the University of Toronto (UT) (funded by Wellcome Trust, Medical Research Council, Canadian Institutes of Health Research), and Genetic Differences (GD) (R01 AG007584). The CHARGE cohorts are supported in part by National Heart, Lung, and Blood Institute (NHLBI) infrastructure grant HL105756 (Psaty), RC2HL102419 (Boerwinkle) and the neurology working group is supported by the National Institute on Aging (NIA) R01 grant AG033193.

The CHARGE cohorts participating in the ADSP include the following: Austrian Stroke Prevention Study (ASPS), ASPS-Family study, and the Prospective Dementia Registry-Austria (ASPS/PRODEM-Aus), the Atherosclerosis Risk in Communities (ARIC) Study, the Cardiovascular Health Study (CHS), the Erasmus Rucphen Family Study (ERF), the Framingham Heart Study (FHS), and the Rotterdam Study (RS). ASPS is funded by the Austrian Science Fond (FWF) grant number P20545-P05 and P13180 and the Medical University of Graz. The ASPS-Fam is funded by the Austrian Science Fund (FWF) project I904), the EU Joint Programme—Neurodegenerative Disease Research (JPND) in frame of the BRIDGET project (Austria, Ministry of Science) and the Medical University of Graz and the Steiermärkische Krankenanstalten Gesellschaft. PRODEM-Austria is supported by the Austrian Research Promotion agency (FFG) (Project No. 827462) and by the Austrian National Bank (Anniversary Fund, project 15435. ARIC research is carried out as a collaborative study supported by NHLBI contracts (HHSN268201100005C, HHSN268201100006C, HHSN268201100007C, HHSN268201100008C, HHSN268201100009C, HHSN268201100010C, HHSN268201100011C, and HHSN268201100012C). Neurocognitive data in ARIC is collected by U01 2U01HL096812, 2U01HL096814, 2U01HL096899, 2U01HL096902, 2U01HL096917 from the NIH (NHLBI, NINDS, NIA and NIDCD), and with previous brain MRI examinations funded by R01-HL70825 from the NHLBI. CHS research was supported by contracts HHSN268201200036C, HHSN268200800007C, N01HC55222, N01HC85079, N01HC85080, N01HC85081, N01HC85082, N01HC85083, N01HC85086, and grants U01HL080295 and U01HL130114 from the NHLBI with additional contribution from the National Institute of Neurological Disorders and Stroke (NINDS). Additional support was provided by R01AG023629, R01AG15928, and R01AG20098 from the NIA. FHS research is supported by NHLBI contracts N01-HC-25195 and HHSN268201500001I. This study was also supported by additional grants from the NIA (R01s AG054076, AG049607 and AG033040 and NINDS (R01 NS017950). The ERF study as a part of EUROSPAN (European Special Populations Research Network) was supported by European Commission FP6 STRP grant number 018947 (LSHG-CT-2006-01947) and also received funding from the European Community’s Seventh Framework Programme (FP7/2007 to 2013)/grant agreement HEALTH-F4- 2007-201413 by the European Commission under the programme “Quality of Life and Management of the Living Resources” of 5th Framework Programme (no. QLG2-CT-2002- 01254). High-throughput analysis of the ERF data was supported by a joint grant from the Netherlands Organization for Scientific Research and the Russian Foundation for Basic Research (NWO-RFBR 047.017.043). The Rotterdam Study is funded by Erasmus Medical Center and Erasmus University, Rotterdam, the Netherlands Organization for Health Research and Development (ZonMw), the Research Institute for Diseases in the Elderly (RIDE), the Ministry of Education, Culture and Science, the Ministry for Health, Welfare and Sports, the European Commission (DG XII), and the municipality of Rotterdam. Genetic data sets are also supported by the Netherlands Organization of Scientific Research NWO Investments (175.010.2005.011, 911-03-012), the Genetic Laboratory of the Department of Internal Medicine, Erasmus MC, the Research Institute for Diseases in the Elderly (014 to 93-015; RIDE2), and the Netherlands Genomics Initiative (NGI)/Netherlands Organization for Scientific Research (NWO) Netherlands Consortium for Healthy Aging (NCHA), project 050 to 060-810. All studies are grateful to their participants, faculty and staff. The content of these manuscripts is solely the responsibility of the authors and does not necessarily represent the official views of the National Institutes of Health or the U.S. Department of Health and Human Services.

The FUS cohorts include: the Alzheimer’s Disease Research Centers (ADRC) (P30 AG062429, P30 AG066468, P30 AG062421, P30 AG066509, P30 AG066514, P30 AG066530, P30 AG066507, P30 AG066444, P30 AG066518, P30 AG066512, P30 AG066462, P30 AG072979, P30 AG072972, P30 AG072976, P30 AG072975, P30 AG072978, P30 AG072977, P30 AG066519, P30 AG062677, P30 AG079280, P30 AG062422, P30 AG066511, P30 AG072946, P30 AG062715, P30 AG072973, P30 AG066506, P30 AG066508, P30 AG066515, P30 AG072947, P30 AG072931, P30 AG066546, P20 AG068024, P20 AG068053, P20 AG068077, P20 AG068082, P30 AG072958, P30 AG072959), Alzheimer’s Disease Neuroimaging Initiative (ADNI) (U19AG024904), Amish Protective Variant Study (RF1AG058066), Cache County Study (R01AG11380, R01AG031272, R01AG21136, RF1AG054052), Case Western Reserve University Brain Bank (CWRUBB) (P50AG008012), Case Western Reserve University Rapid Decline (CWRURD) (RF1AG058267, NU38CK000480), Cuban American Alzheimer’s Disease Initiative (CuAADI) (3U01AG052410), Estudio Familiar de Influencia Genetica en Alzheimer (EFIGA) (5R37AG015473, RF1AG015473, R56AG051876), Genetic and Environmental Risk Factors for Alzheimer Disease Among African Americans Study (GenerAAtions) (2R01AG09029, R01AG025259, 2R01AG048927), Gwangju Alzheimer and Related Dementias Study (GARD) (U01AG062602), Hillblom Aging Network (2014-A-004-NET, R01AG032289, R01AG048234), Hussman Institute for Human Genomics Brain Bank (HIHGBB) (R01AG027944, Alzheimer’s Association “Identification of Rare Variants in Alzheimer Disease”), Ibadan Study of Aging (IBADAN) (5R01AG009956), Longevity Genes Project (LGP) and LonGenity (R01AG042188, R01AG044829, R01AG046949, R01AG057909, R01AG061155, P30AG038072), Mexican Health and Aging Study (MHAS) (R01AG018016), Multi-Institutional Research in Alzheimer’s Genetic Epidemiology (MIRAGE) (2R01AG09029, R01AG025259, 2R01AG048927), Northern Manhattan Study (NOMAS) (R01NS29993), Peru Alzheimer’s Disease Initiative (PeADI) (RF1AG054074), Puerto Rican 1066 (PR1066) (Wellcome Trust (GR066133/GR080002), European Research Council (340755)), Puerto Rican Alzheimer Disease Initiative (PRADI) (RF1AG054074), Reasons for Geographic and Racial Differences in Stroke (REGARDS) (U01NS041588), Research in African American Alzheimer Disease Initiative (REAAADI) (U01AG052410), the Religious Orders Study (ROS) (P30 AG10161, P30 AG72975, R01 AG15819, R01 AG42210), the RUSH Memory and Aging Project (MAP) (R01 AG017917, R01 AG42210Stanford Extreme Phenotypes in ad (R01AG060747), University of Miami Brain Endowment Bank (MBB), University of Miami/Case Western/North Carolina A&T African American (UM/CASE/NCAT) (U01AG052410, R01AG028786), Wisconsin Registry for Alzheimer’s Prevention (WRAP) (R01AG027161 and R01AG054047), Mexico-Southern California Autosomal Dominant Alzheimer’s Disease Consortium (R01AG069013), Center for Cognitive Neuroscience and Aging (R01AG047649), and the A4 Study (R01AG063689, U19AG010483 and U24AG057437).

The four LSACs are: the Human Genome Sequencing Center at the Baylor College of Medicine (U54 HG003273), the Broad Institute Genome Center (U54HG003067), The American Genome Center at the Uniformed Services University of the Health Sciences (U01AG057659), and the Washington University Genome Institute (U54HG003079). Genotyping and sequencing for the ADSP FUS is also conducted at John P. Hussman Institute for Human Genomics (HIHG) Center for Genome Technology (CGT).

Biological samples and associated phenotypic data used in primary data analyses were stored at Study Investigators institutions, and at the National Centralized Repository for Alzheimer’s Disease and Related Dementias (NCRAD, U24AG021886) at Indiana University funded by NIA. Associated Phenotypic Data used in primary and secondary data analyses were provided by Study Investigators, the NIA funded Alzheimer’s Disease Centers (ADCs), and the National Alzheimer’s Coordinating Center (NACC, U24AG072122) and the National Institute on Aging Genetics of Alzheimer’s Disease Data Storage Site (NIAGADS, U24AG041689) at the University of Pennsylvania, funded by NIA. Harmonized phenotypes were provided by the ADSP Phenotype Harmonization Consortium (ADSP-PHC), funded by NIA (U24 AG074855, U01 AG068057 and R01 AG059716) and Ultrascale Machine Learning to Empower Discovery in Alzheimer’s Disease Biobanks (AI4AD, U01 AG068057). This research was supported in part by the Intramural Research Program of the National Institutes of health, National Library of Medicine. Contributors to the Genetic Analysis Data included Study Investigators on projects that were individually funded by NIA, and other NIH institutes, and by private U.S. organizations, or foreign governmental or nongovernmental organizations.

The ADSP Phenotype Harmonization Consortium (ADSP-PHC) is funded by NIA (U24 AG074855, U01 AG068057 and R01 AG059716). The harmonized cohorts within the ADSP-PHC include: the Anti-Amyloid Treatment in Asymptomatic Alzheimer’s study (A4 Study), a secondary prevention trial in preclinical Alzheimer’s disease, aiming to slow cognitive decline associated with brain amyloid accumulation in clinically normal older individuals. The A4 Study is funded by a public-private-philanthropic partnership, including funding from the National Institutes of Health-National Institute on Aging, Eli Lilly and Company, Alzheimer’s Association, Accelerating Medicines Partnership, GHR Foundation, an anonymous foundation and additional private donors, with in-kind support from Avid and Cogstate. The companion observational Longitudinal Evaluation of Amyloid Risk and Neurodegeneration (LEARN) Study is funded by the Alzheimer’s Association and GHR Foundation. The A4 and LEARN Studies are led by Dr Reisa Sperling at Brigham and Women’s Hospital, Harvard Medical School and Dr Paul Aisen at the Alzheimer’s Therapeutic Research Institute (ATRI), University of Southern California. The A4 and LEARN Studies are coordinated by ATRI at the University of Southern California, and the data are made available through the Laboratory for Neuro Imaging at the University of Southern California. The participants screening for the A4 Study provided permission to share their de-identified data in order to advance the quest to find a successful treatment for Alzheimer’s disease. We would like to acknowledge the dedication of all the participants, the site personnel, and all of the partnership team members who continue to make the A4 and LEARN Studies possible. The complete A4 Study Team list is available on: a4study.org/a4-study-team.; the Adult Changes in Thought study (ACT), U01 AG006781, U19 AG066567; Alzheimer’s Disease Neuroimaging Initiative (ADNI): Data collection and sharing for this project was funded by the Alzheimer’s Disease Neuroimaging Initiative (ADNI) (National Institutes of Health Grant U01 AG024904) and DOD ADNI (Department of Defense award number W81XWH-12-2-0012). ADNI is funded by the National Institute on Aging, the National Institute of Biomedical Imaging and Bioengineering, and through generous contributions from the following: AbbVie, Alzheimer’s Association; Alzheimer’s Drug Discovery Foundation; Araclon Biotech; BioClinica, Inc.; Biogen; Bristol-Myers Squibb Company; CereSpir, Inc.; Cogstate; Eisai Inc.; Elan Pharmaceuticals, Inc.; Eli Lilly and Company; EuroImmun; F. Hoffmann-La Roche Ltd and its affiliated company Genentech, Inc.; Fujirebio; GE Healthcare; IXICO Ltd; Janssen Alzheimer Immunotherapy Research & Development, LLC.; Johnson & Johnson Pharmaceutical Research & Development LLC.; Lumosity; Lundbeck; Merck & Co., Inc.; Meso Scale Diagnostics, LLC.; NeuroRx Research; Neurotrack Technologies; Novartis Pharmaceuticals Corporation; Pfizer Inc.; Piramal Imaging; Servier; Takeda Pharmaceutical Company; and Transition Therapeutics. The Canadian Institutes of Health Research is providing funds to support ADNI clinical sites in Canada. Private sector contributions are facilitated by the Foundation for the National Institutes of Health (www.fnih.org). The grantee organization is the Northern California Institute for Research and Education, and the study is coordinated by the Alzheimer’s Therapeutic Research Institute at the University of Southern California. ADNI data are disseminated by the Laboratory for Neuro Imaging at the University of Southern California; Estudio Familiar de Influencia Genetica en Alzheimer (EFIGA): 5R37AG015473, RF1AG015473, R56AG051876; the Health & Aging Brain Study—Health Disparities (HABS-HD), supported by the National Institute on Aging of the National Institutes of Health under Award Numbers R01AG054073, R01AG058533, R01AG070862, P41EB015922, and U19AG078109; the Korean Brain Aging Study for the Early Diagnosis and Prediction of Alzheimer’s disease (KBASE), which was supported by a grant from Ministry of Science, ICT and Future Planning (Grant No: NRF-2014M3C7A1046042); Memory & Aging Project at Knight Alzheimer’s Disease Research Center (MAP at Knight ADRC): The Memory and Aging Project at the Knight-ADRC (Knight-ADRC). This work was supported by the National Institutes of Health (NIH) grants R01AG064614, R01AG044546, RF1AG053303, RF1AG058501, U01AG058922 and R01AG064877 to Carlos Cruchaga. The recruitment and clinical characterization of research participants at Washington University was supported by NIH grants P30AG066444, P01AG03991, and P01AG026276. Data collection and sharing for this project was supported by NIH grants RF1AG054080, P30AG066462, R01AG064614 and U01AG052410. We thank the contributors who collected samples used in this study, as well as patients and their families, whose help and participation made this work possible. This work was supported by access to equipment made possible by the Hope Center for Neurological Disorders, the Neurogenomics and Informatics Center (NGI: https://neurogenomics.wustl.edu/) and the Departments of Neurology and Psychiatry at Washington University School of Medicine; National Alzheimer’s Coordinating Center (NACC): The NACC database is funded by NIA/NIH Grant U24 AG072122. SCAN is a multi-institutional project that was funded as a U24 grant (AG067418) by the National Institute on Aging in May 2020. Data collected by SCAN and shared by NACC are contributed by the NIA-funded ADRCs as follows: P30 AG062429 (PI James Brewer, MD, PhD), P30 AG066468 (PI Oscar Lopez, MD), P30 AG062421 (PI Bradley Hyman, MD, PhD), P30 AG066509 (PI Thomas Grabowski, MD), P30 AG066514 (PI Mary Sano, PhD), P30 AG066530 (PI Helena Chui, MD), P30 AG066507 (PI Marilyn Albert, PhD), P30 AG066444 (PI John Morris, MD), P30 AG066518 (PI Jeffrey Kaye, MD), P30 AG066512 (PI Thomas Wisniewski, MD), P30 AG066462 (PI Scott Small, MD), P30 AG072979 (PI David Wolk, MD), P30 AG072972 (PI Charles DeCarli, MD), P30 AG072976 (PI Andrew Saykin, PsyD), P30 AG072975 (PI David Bennett, MD), P30 AG072978 (PI Neil Kowall, MD), P30 AG072977 (PI Robert Vassar, PhD), P30 AG066519 (PI Frank LaFerla, PhD), P30 AG062677 (PI Ronald Petersen, MD, PhD), P30 AG079280 (PI Eric Reiman, MD), P30 AG062422 (PI Gil Rabinovici, MD), P30 AG066511 (PI Allan Levey, MD, PhD), P30 AG072946 (PI Linda Van Eldik, PhD), P30 AG062715 (PI Sanjay Asthana, MD, FRCP), P30 AG072973 (PI Russell Swerdlow, MD), P30 AG066506 (PI Todd Golde, MD, PhD), P30 AG066508 (PI Stephen Strittmatter, MD, PhD), P30 AG066515 (PI Victor Henderson, MD, MS), P30 AG072947 (PI Suzanne Craft, PhD), P30 AG072931 (PI Henry Paulson, MD, PhD), P30 AG066546 (PI Sudha Seshadri, MD), P20 AG068024 (PI Erik Roberson, MD, PhD), P20 AG068053 (PI Justin Miller, PhD), P20 AG068077 (PI Gary Rosenberg, MD), P20 AG068082 (PI Angela Jefferson, PhD), P30 AG072958 (PI Heather Whitson, MD), P30 AG072959 (PI James Leverenz, MD); National Institute on Aging Alzheimer’s Disease Family Based Study (NIA-ad FBS): U24 AG056270; Religious Orders Study (ROS): P30AG10161, R01AG15819, R01AG42210; Memory and Aging Project (MAP—Rush): R01AG017917, R01AG42210; Minority Aging Research Study (MARS): R01AG22018, R01AG42210; the Texas Alzheimer’s Research and Care Consortium (TARCC), funded by the Darrell K Royal Texas Alzheimer’s Initiative, directed by the Texas Council on Alzheimer’s Disease and Related Disorders; Washington Heights/Inwood Columbia Aging Project (WHICAP): RF1 AG054023; and Wisconsin Registry for Alzheimer’s Prevention (WRAP): R01AG027161 and R01AG054047. Additional acknowledgments include the National Institute on Aging Genetics of Alzheimer’s Disease Data Storage Site (NIAGADS, U24AG041689) at the University of Pennsylvania, funded by NIA.

## NACC

The NACC database is funded by NIA/NIH Grant U24 AG072122. NACC data are contributed by the NIA-funded ADRCs: P30 AG062429 (PI James Brewer, MD, PhD), P30 AG066468 (PI Oscar Lopez, MD), P30 AG062421 (PI Bradley Hyman, MD, PhD), P30 AG066509 (PI Thomas Grabowski, MD), P30 AG066514 (PI Mary Sano, PhD), P30 AG066530 (PI Helena Chui, MD), P30 AG066507 (PI Marilyn Albert, PhD), P30 AG066444 (PI David Holtzman, MD), P30 AG066518 (PI Lisa Silbert, MD, MCR), P30 AG066512 (PI Thomas Wisniewski, MD), P30 AG066462 (PI Scott Small, MD), P30 AG072979 (PI David Wolk, MD), P30 AG072972 (PI Charles DeCarli, MD), P30 AG072976 (PI Andrew Saykin, PsyD), P30 AG072975 (PI Julie A. Schneider, MD, MS), P30 AG072978 (PI Ann McKee, MD), P30 AG072977 (PI Robert Vassar, PhD), P30 AG066519 (PI Frank LaFerla, PhD), P30 AG062677 (PI Ronald Petersen, MD, PhD), P30 AG079280 (PI Jessica Langbaum, PhD), P30 AG062422 (PI Gil Rabinovici, MD), P30 AG066511 (PI Allan Levey, MD, PhD), P30 AG072946 (PI Linda Van Eldik, PhD), P30 AG062715 (PI Sanjay Asthana, MD, FRCP), P30 AG072973 (PI Russell Swerdlow, MD), P30 AG066506 (PI Glenn Smith, PhD, ABPP), P30 AG066508 (PI Stephen Strittmatter, MD, PhD), P30 AG066515 (PI Victor Henderson, MD, MS), P30 AG072947 (PI Suzanne Craft, PhD), P30 AG072931 (PI Henry Paulson, MD, PhD), P30 AG066546 (PI Sudha Seshadri, MD), P30 AG086401 (PI Erik Roberson, MD, PhD), P30 AG086404 (PI Gary Rosenberg, MD), P20 AG068082 (PI Angela Jefferson, PhD), P30 AG072958 (PI Heather Whitson, MD), P30 AG072959 (PI James Leverenz, MD).
