# Supplementary material for: GRN rs5848 variant associates with TDP-43 pathology and cancer in opposite directions
Source: J Neuropathol Exp Neurol. 2026 May 21;85(9):1003–19. doi: 10.1093/jnen/nlag051 (PMC13314055; doi:10.1093/jnen/nlag051)
Supplement: Supplementary file 1 [file nlag051_supplementary_data.docx]

**Supplementary Figure 1**. UMAP projection of the Alzheimer's Disease Sequencing Project (ADSP) samples based on genome-wide common variants, colored by predicted super-population assignment. Clustering recapitulates major continental ancestries, including European (EUR), African (AFR), Admixed American (AMR), East Asian (EAS), and South Asian (SAS). The process was repeated for samples from the Alzheimer’s Disease Genetics Consortium (ADGC).

**Supplementary Table 1**. Exclusion criteria in the National Alzheimer’s Coordination Center Neuropathology Form

| Exclusion criteria | Variable name |
| --- | --- |
| Down syndrome | NACCDOWN |
| Pigment-spheroid degeneration/NBIA | NPPDXA |
| Multiple system atrophy | NPPDXB |
| Trinucleotide disease (Huntington disease, SCA, other) | NPPDXD |
| Malformation of cortical development | NPPDXE |
| Metabolic/storage disorder of any type | NPPDXF |
| White matter disease, leukodystrophy | NPPDXG |
| White matter disease, multiple sclerosis or other demyelinating disease | NPPDXH |
| Prion disease | NACCPRIO |
| FTLD-tau | NPFTDTAU |
| ALS/motor neuron disease (MND) | NPALSMND |
| CADASIL | NPPATH10 |
| FTLD with TDP-43 pathology (FTLD-TDP) | NPFTDTDP |
| Other FTLD | NPOFTD |
| FTLD-tau subtype – Pick’s (PiD) | NACCPICK |
| FTLD-tau subtype — other 3R tauopathy (includes MAPT mutation tauopathy) | NPFTDT2 |
| FTLD-tau subtype — corticobasal degeneration (CBD) | NACCCBD |
| FTLD-tau subtype — progressive supranuclear palsy (PSP) | NACCPROG |
| FTLD-tau subtype — argyrophilic grains | NPFTDT5 |
| FTLD-tau subtype — other 4R tauopathy (includes sporadic multiple systems  tauopathy, globular glial tauopathy, MAPT mutation tauopathy) | NPFTDT6 |
| FTLD-tau subtype — chronic traumatic encephalopathy | NPFTDT7 |
| FTLD-tau subtype — amyotrophic lateral sclerosis (ALS)/parkinsonism-dementia | NPFTDT8 |
| FTLD-tau subtype — tangle dominant disease | NPFTDT9 |
| FTLD-tau subtype — other 3R + 4R tauopathy | NPFTDT10 |
| Frontemporal dementia and parkinsonism with tau-positive or argyrophilic inclusions | NPFRONT |
| Tauopathy, other (e.g., tangle-only dementia and argyrophilic grain dementia) | NPTAU |
| FTD with ubiquitin-positive (tau-negative) inclusions | NPFTD |

**Supplementary Table 2**. Frequency of rs5848 T-allele carriers across major cancer types in ADSP-NACC and ADGC-NACC cohorts

| Cancer type | ADSP-NACC | |  | ADGC-NACC | |
| --- | --- | --- | --- | --- | --- |
|  | n | rs5848 T carriers  n (%) |  | n | rs5848 T carriers  n (%) |
| Breast | 155 | 80 (51.6) |  | 365 | 151 (41.4) |
| Prostate | 138 | 61 (44.2) |  | 421 | 205 (48.7) |
| Melanoma | 77 | 37 (48.1) |  | 206 | 102 (49.5) |
| Colon | 33 | 11 (33.3) |  | 84 | 37 (44.0) |
| Lung | 25 | 6 (24.0) |  | 56 | 31 (55.4) |

**Supplementary Table 3.** Associations of *GRN* rs5848, *TMEM106B* rs1990622, *BIN1* risk variants (rs744373, rs6733839), and *APOE ε4* with cancer history and neuropathologic outcomes across the ADGC-NACC and ADSP-NACC cohorts and in fixed-effect meta-analysis.

| Cohort | Phenotype | rsID | Effect allele | Non-effect allele | OR | Lower  95% CI | Upper  95% CI | P value |
| --- | --- | --- | --- | --- | --- | --- | --- | --- |
| Meta-analysis | Cancer status | rs5849 | T | C | 0.906 | 0.838 | 0.979 | 0.012 |
| Meta-analysis | HS-Aging | rs5848 | T | C | 1.504 | 1.299 | 1.742 | 4.9×10^-8^ |
| Meta-analysis | TDP-43 in hippocampus | rs5848 | T | C | 1.421 | 1.197 | 1.686 | 5.9×0^-5^ |
| Meta-analysis | TDP-43 in amygdala | rs5848 | T | C | 1.155 | 0.986 | 1.353 | 0.075 |
| Meta-analysis | TDP-43 in neocortex | rs5848 | T | C | 1.973 | 1.455 | 2.677 | 1.2×10^-5^ |
| ADSP-NACC | HS-Aging | rs5848 | T | C | 1.590 | 1.273 | 1.984 | 4.2×10^-5^ |
| ADSP-NACC | TDP-43 in hippocampus | rs5848 | T | C | 1.289 | 0.982 | 1.691 | 0.067 |
| ADSP-NACC | TDP-43 in amygdala | rs5848 | T | C | 1.128 | 0.889 | 1.432 | 0.322 |
| ADSP-NACC | TDP-43 in neocortex | rs5848 | T | C | 1.584 | 0.961 | 2.611 | 0.071 |
| ADSP-NACC | Cancer status | rs5849 | T | C | 0.881 | 0.760 | 1.020 | 0.090 |
| ADGC-NACC | HS-Aging | rs5848 | T | C | 1.441 | 1.185 | 1.752 | 2.5×10^-4^ |
| ADGC-NACC | TDP-43 in hippocampus | rs5848 | T | C | 1.516 | 1.215 | 1.890 | 2.2 ×10^-4^ |
| ADGC-NACC | TDP-43 in amygdala | rs5848 | T | C | 1.176 | 0.952 | 1.453 | 0.133 |
| ADGC-NACC | TDP-43 in neocortex | rs5848 | T | C | 2.248 | 1.530 | 3.304 | 3.7 ×10^-5^ |
| ADGC-NACC | Cancer status | rs5848 | T | C | 0.916 | 0.836 | 1.003 | 0.057 |
| Meta-analysis | Cancer status | rs744373 | G | A | 0.977 | 0.916 | 1.041 | 0.473 |
| Meta-analysis | Cancer status | rs6733839 | T | C | 0.943 | 0.887 | 1.002 | 0.058 |
| Meta-analysis | Braak NFT stage | rs744373 | G | A | 1.193 | 1.088 | 1.308 | 1.7 ×10^-4^ |
| Meta-analysis | Braak NFT stage | rs6733839 | T | C | 1.176 | 1.078 | 1.283 | 2.6 ×10^-4^ |
| Meta-analysis | CERAD | rs744373 | G | A | 1.201 | 1.086 | 1.329 | 3.8×10^-4^ |
| Meta-analysis | CERAD | rs6733839 | T | C | 1.187 | 1.079 | 1.306 | 4.5×10^-4^ |

**Supplementary Table 3.** (Continued)

| Cohort | Phenotype | Genetic Marker | Effect allele | Non-effect allele | OR | Lower  95% CI | Upper  95% CI | P value |
| --- | --- | --- | --- | --- | --- | --- | --- | --- |
| ADSP-NACC | Cancer status | rs6733839 | T | C | 0.938 | 0.836 | 1.052 | 0.273 |
| ADSP-NACC | Cancer status | rs744373 | G | A | 1.033 | 0.914 | 1.168 | 0.602 |
| ADSP-NACC | Braak NFT stage | rs6733839 | T | C | 1.298 | 1.117 | 1.509 | 0.001 |
| ADSP-NACC | Braak NFT stage | rs744373 | G | A | 1.272 | 1.085 | 1.491 | 0.003 |
| ADSP-NACC | CERAD SCORE | rs6733839 | T | C | 1.396 | 1.177 | 1.656 | 1.2 ×10^-4^ |
| ADSP-NACC | CERAD | rs744373 | G | A | 1.305 | 1.090 | 1.562 | 3.7 ×10^-3^ |
| ADGC-NACC | Cancer status | rs6733839 | T | C | 0.945 | 0.879 | 1.015 | 0.121 |
| ADGC-NACC | Cancer status | rs744373 | G | A | 0.956 | 0.887 | 1.031 | 0.245 |
| ADGC-NACC | Braak NFT stage | rs6733839 | T | C | 1.119 | 1.006 | 1.245 | 0.038 |
| ADGC-NACC | Braak NFT stage | rs744373 | G | A | 1.155 | 1.032 | 1.293 | 0.012 |
| ADGC-NACC | CERAD | rs6733839 | T | C | 1.102 | 0.982 | 1.237 | 0.0997 |
| ADGC-NACC | CERAD | rs744373 | G | A | 1.156 | 1.023 | 1.306 | 0.020 |
| Meta-analysis | Cancer status | *APOE ε4* | A | C | 0.950 | 0.885 | 1.019 | 0.152 |
| Meta-analysis | ADNC | *APOE ε4* | A | C | 2.649 | 2.297 | 3.054 | 4.8×10^-41^ |
| ADSP-NACC | Cancer status | *APOE ε4* | A | C | 1.001 | 0.881 | 1.138 | 0.987 |
| ADSP-NACC | ADNC | *APOE ε4* | A | C | 2.078 | 1.638 | 2.636 | 1.6×10^-9^ |
| ADGC-NACC | Cancer status | *APOE ε4* | A | C | 0.929 | 0.854 | 1.010 | 0.085 |
| ADGC-NACC | ADNC | *APOE ε4* | A | C | 3.033 | 2.539 | 3.622 | 2×10^−34^ |
| Meta-analysis | Cancer status | rs1990622 | A | G | 0.986 | 0.929 | 1.048 | 0.659 |
| Meta-analysis | HS-Aging | rs1990622 | A | G | 1.768 | 1.530 | 2.042 | 1×10^-14^ |
| Meta-analysis | TDP-43 in hippocampus | rs1990622 | A | G | 1.672 | 1.429 | 1.956 | 1.4×10^-10^ |
| Meta-analysis | TDP-43 in amygdala | rs1990622 | A | G | 1.473 | 1.261 | 1.721 | 1.0×10^-6^ |
| Meta-analysis | TDP-43 in neocortex | rs1990622 | A | G | 1.561 | 1.155 | 2.111 | 0.0038 |

**Supplementary Table 3.** (Continued)

| Cohort | Phenotype | Genetic Marker | Effect allele | Non-Effect allele | OR | Lower  95% CI | Upper  95% CI | P value |
| --- | --- | --- | --- | --- | --- | --- | --- | --- |
| ADSP-NACC | Cancer status | rs1990622 | A | G | 0.901 | 0.803 | 1.010 | 0.073 |
| ADSP-NACC | HS-Aging | rs1990622 | A | G | 1.747 | 1.401 | 2.178 | 7.2×10^-7^ |
| ADSP-NACC | TDP-43 in hippocampus | rs1990622 | A | G | 1.469 | 1.163 | 1.856 | 0.0012 |
| ADSP-NACC | TDP-43 in amygdala | rs1990622 | A | G | 1.357 | 1.071 | 1.719 | 0.011 |
| ADSP-NACC | TDP-43 in neocortex | rs1990622 | A | G | 1.209 | 0.771 | 1.898 | 0.408 |
| ADGC-NACC | Cancer status | rs1990622 | A | G | 1.022 | 0.952 | 1.096 | 0.555 |
| ADGC-NACC | HS-Aging | rs1990622 | A | G | 1.783 | 1.474 | 2.158 | 2.8×10^-9^ |
| ADGC-NACC | TDP-43 in hippocampus | rs1990622 | A | G | 1.859 | 1.504 | 2.298 | 9.7×10 ^-9^ |
| ADGC-NACC | TDP-43 in amygdala | rs1990622 | A | G | 1.568 | 1.276 | 1.927 | 1.9×10^-5^ |
| ADGC-NACC | TDP-43 in neocortex | rs1990622 | A | G | 1.920 | 1.280 | 2.882 | 0.0016 |

ADNC: NIA-AA Alzheimer’s disease neuropathologic change, ADSP: the Alzheimer’s Disease Sequencing Project, ADGC: Alzheimer's Disease Genetics Consortium, NACC: National Alzheimer's Coordinating Center, HS aging: Hippocampal sclerosis of Aging, including mesial temporal sclerosis, rsID : reference SNP ID, OR: odds ratio, CI: confidence interval, Braak NFT: Braak neurofibrillary tangle stage, CERAD score: Consortium to Establish a Registry for Alzheimer’s Disease neuritic plaque score.

Neuropathologic outcomes (CERAD score, Braak NFT stage) were analyzed as ordinal traits using proportional odds logistic mixed models, whereas cancer status, TDP-43 pathology and HS aging were analyzed as a binary outcome using Scalable and Accurate Implementation of Generalized Linear Mixed Models (SAIGE) logistic mixed models. All models were adjusted for sex, principal components of ancestry, and age. Age at death was used as an age covariate for neuropathologic outcomes in genetic analyses, while age at last clinical assessment was used for cancer outcomes, with cancer defined as having been reported at least once during follow-up.

**Supplementary Table 4**. Association of rs5848 T allele with breast and prostate cancers in ADSP-NACC and ADGC-NACC cohorts, with meta-analysis

| Cancer type | OR (95% CI) | | |
| --- | --- | --- | --- |
|  | ADSP-NACC | ADGC-NACC | Meta-analysis |
| Breast | 1.08 (0.83-1.40) | 0.77 (0.63-0.93) | 0.87 (0.74-1.02) |
| Prostate | 0.79 (0.59-1.05) | 0.93 (0.79-1.10) | 0.89 (0.77-1.03) |

OR: odds ratio, CI: confidence interval

**Supplementary Table 5**. Demographic and neuropathological characteristics of ADSP-NACC and ADGC-NACC participants with complete data on TDP-43 pathology and cancer history with genetic data

| Characteristics  n (%) or mean ± SD | ADSP-NACC | | |  | ADGC-NACC | | |
| --- | --- | --- | --- | --- | --- | --- | --- |
|  | NP available^*^ | No cancer | Cancer cases |  | NP available^*^ | No cancer | Cancer cases |
|  | (n = 588) | (n = 427) | (n = 161) |  | (n = 835) | (n = 619) | (n = 216) |
| Age at death | 79.6 ± 10.7 | 78.1 ± 10.9 | 84.1 ± 8.6 |  | 83.9 ± 10.3 | 83.3 ± 10.9 | 85.3 ± 8.7 |
| TDP-43 pathology |  |  |  |  |  |  |  |
| Amygdala | 203 (37.2) | 155 (37.3) | 48 (36.6) |  | 263 (33.0) | 184 (33.3) | 79 (32.2) |
| Hippocampus | 161 (27.4) | 124 (27.7) | 37 (26.2) |  | 216 (25.9) | 157 (27.2) | 59 (23.0) |
| Neocortex | 30 (5.7) | 25 (6.3) | 5 (3.9) |  | 46 (6.1) | 34 (6.6) | 12 (5.1) |
| LATE stage ≥2 | 154 (30.8) | 116 (30.8) | 38 (30.9) |  | 196 (26.8) | 143 (28.4) | 53 (23.5) |
| rs5848 T allele carrier | 328 (55.8) | 251 (56.2) | 77 (54.6) |  | 425 (50.9) | 301 (52.1) | 124 (48.2) |

*: participants with complete data on TDP-43 pathology in hippocampus and cancer history with genetic data

**Supplementary Table 6**. Interaction effect between cancer history and rs5848 T allele on LATE-NC stages in combined ADSP-NACC and ADGC-NACC cohorts (n = 1,423)

| Variable | $\hat{\beta}$ | SE | P value |
| --- | --- | --- | --- |
| Cancer history | -0.001 | 0.18 | >0.99 |
| rs5848 T allele Carrier | 0.407 | 0.10 | 0.0030 |
| Cancer × rs5848 T interaction | -0.027 | 0.12 | 0.82 |

SE: Standard error

Adjusted for age at death, sex, and cohorts (ADSP-NACC or ADGC-NACC).

**Supplementary Table 7**. *GRN*/PGRN and rs5848 in TDP-43–related neurodegeneration

| Author (Year) | Disease | PMID | Gene/Protein/Variant | Key Findings |
| --- | --- | --- | --- | --- |
| Nelson PT et al.  (2024) | LATE-NC | 38613823 | rs5848 | *GRN* identified as a genetic risk factor linked to TDP-43–related neurodegeneration. |
| Katsumata Y et al.  (2024) | Dementia‐related proteinopathies | 38460116 | rs5848 | rs5848 T allele is associated with for TDP‐43 pathology. |
| Fardo DW et al.  (2017) | HS-Aging/LATE-NC | 28189700 | rs5848 | rs5848 T allele is associated with altered CSF protein profiles, including increased tau and inflammatory markers. |
| Dickson DW et al.  (2010) | Hippocampal sclerosis | [20197700](https://pubmed.ncbi.nlm.nih.gov/20197700/) | rs5848 | Common variant in *GRN* is a genetic risk factor for hippocampal sclerosis in the elderly. |
| Gass J et al.  (2006) | FTLD | 16950801 | rs5848 | Mutations in progranulin are a major cause of ubiquitin-positive frontotemporal lobar degeneration. |
| Hsiung G et al.  (2010) | FTD and possibly other dementias | 21047645 | rs5848 | rs5848 T allele associated with reduced serum PGRN levels. |
| Fenoglio C et al.  (2009) | AD | 38037070 | rs5848 | rs5848 genotype influences *GRN* mRNA levels in brain and peripheral cells, supporting functional regulation of progranulin expression. |
| Pickering-Brown SM et al. (2008) | FTLD | 18192287 | *GRN* variants | *GRN* mutations identified in FTLD patients with distinct clinical phenotypes and variable family history |
| Yang HS et al.  (2020) | TDP-43 proteinopathy | [32526197](https://pubmed.ncbi.nlm.nih.gov/32526197/) | *GRN* variants | *GRN* variants show coordinated effects on aging brain transcriptome. |

**Supplementary Table 7**. (continued)

| Author (Year) | Disease | PMID | Gene/Protein/Variant | Key Findings |
| --- | --- | --- | --- | --- |
| Rademakers R et al.  (2008) | TDP 43-positive FTD | 18723524 | rs5848 | rs5848 T allele associated with reduced GRN protein levels and TDP-43–positive neuropathology (biochemical and IHC evidence) |
| Chen Y et al.  (2015) | Neurodegeneration (FTLD, AD, PD, ALS) | 25578179 | rs5848 | rs5848 T allele is associated with increased risk of multiple neurodegenerative diseases, including AD and PD |
| Mateo I et al.  (2013) | PD | 23398167 | PGRN/rs5848 | Reduced serum progranulin levels are associated with Parkinson’s disease risk, independent of rs5848 genotype |
| Cruts M et al.  (2006) | FTLD-TDP | 16862115 | PGRN/*GRN* variants | *GRN* loss-of-function mutations result in reduced PGRN expression. |
| Baker M et al.  (2006) | FTLD-TDP | 16862116 | *GRN* variants | Mutations in progranulin cause tau-negative frontotemporal dementia. |
| Snowden JS et al.  (2006) | Neurodegeneration (FTLD), non-fluent aphasia | 17003069 | *GRN* variants | Progranulin gene mutations associated with frontotemporal dementia and progressive non-fluent aphasia. |
| Reich M et al.  (2024) | FTLD | 38838131 | *GRN* | Peripheral expression of progranulin rescues neurodegenerative pathology |
| Feng T et al.  (2023) | ALS/FTLD | 37519899 | *GRN* | Gene therapy partially rescues motor and pathological phenotypes. |
| Root J et al.  (2024) | Neurodegeneration | 39565694 | PGRN | Restoration rescues inflammation, lysosomal dysfunction, and neuropathology. |
| Bagyinszky E et al.  (2025) | Neurodegeneration (FTLD) | 41155255 | PGRN | Review of therapeutic strategies targeting progranulin deficiency. |

**Supplementary Table 8.** *GRN*/PGRN expression and functional roles in cancer

| Author (Year) | Disease | PMID | Gene/Protein/Variant | Key Findings |
| --- | --- | --- | --- | --- |
| Bandey I et al.  (2015) | Glioblastoma | 24793792 | PGRN | Progranulin promotes temozolomide resistance via DNA repair and tumor stemness. |
| Wang M et al.  (2012) | Glioblastoma | 22161130 | PGRN | Elevated progranulin expressions are associated with poorer survival in glioblastoma patients. |
| Liau LM et al.  (2000) | Glioma | 10728698 | Granulin (PGRN) | Identification of granulin as a glioma-associated growth factor. |
| Wang XW et al.  (2010) | Cancer | 20884628 | PGRN (*GRN*) /miR-15/107 | *GRN* expression is regulated by miR-15/107 microRNAs; dysregulation leads to increased PGRN expression in multiple cancers. |
| Ning X et al.  (2025) | Cancer | 40858049 | PGRN | High expression of PGRN correlated with poor overall survival in human ovarian cancer. |
| Serrero G et al.  (2003) | Breast cancer | 14652816 | PGRN | PGRN is overexpressed in malignant breast tissue and correlates with tumor grade (IHC evidence). |
| Donald CD et al.  (2002) | Renal cell carcinoma | 11911241 | PGRN | Progranulin expression is increased in malignant renal tissue (IHC evidence). |
| Pan CX et al.  (2004) | Prostate cancer | 14977833 | PGRN | Progranulin expression is markedly increased in prostatic intraepithelial neoplasia and invasive prostate cancer (IHC evidence). |
| Cheung ST et al.  (2004) | Hepatocellular carcinoma | 15569995 | PGRN | Progranulin overexpression promotes tumor growth and invasion in hepatocellular carcinoma. |
| Jones MB et al.  (2006) | Endometrial cancer | 16697948 | PGRN | Progranulin acts as a steroid-regulated growth factor in endometrial cancer. |

**Supplementary Table 8.** (continued)

| Author (Year) | Disease | PMID | Gene/Protein/Variant | Key Findings |
| --- | --- | --- | --- | --- |
| Matsumura N et al.  (2006) | Uterine leiomyosarcoma | 16533762 | PGRN | Progranulin exhibits oncogenic properties and contributes to tumorigenesis in vivo. |
| Wang W et al.  (2003) | Multiple myeloma | 12796389 | PGRN | Progranulin promotes proliferation and survival in multiple myeloma. |
| Zhang H et al.  (1998) | Teratoma | [9826678](https://pubmed.ncbi.nlm.nih.gov/9826678/) | PGRN | Suppression of PGRN expression reduces tumorigenicity in teratoma models. |
| Lu R et al.  (2000) | Breast cancer | 10760271 | PGRN | Inhibition of PGRN expression reduces tumor growth in breast cancer cells. |
| Chen XY et al.  (2008) | Esophageal squamous cell carcinoma | 18706200 | PGRN | PGRN expression is associated with tumor progression and clinicopathological features. |
| Rosas I et al.  (2021) | Cancer | 34673649 | rs5848 | genotype TT GRN-rs5848 was statistically more frequent in PD patients without cancer. |
| Tangkeangsirisin W et al.  (2004) | Cancer | 15117809 | PGRN | PGRN promotes migration, invasiveness, angiogenesis, and tumor progression. |
| Jones MB et al.  (2003) | Epithelial ovarian cancer | 12538450 | PGRN | PGRN (GEP/PCDGF) is overexpressed in invasive tumors, promotes proliferation and growth. |
| Monami G et al.  (2006) | Bladder cancer | 16849556 | PGRN | PGRN promotes migration and invasion of tumor. |
| Ho JC et al.  (2008) | Hepatocellular carcinoma | 18393387 | PGRN | PGRN as a therapeutic target. |
